# Supplementary material for: Care and support when a baby is stillborn: A systematic review and an interpretive meta-synthesis of qualitative studies in high-income countries
Source: PLoS One. 2023 Aug 15;18(8):e0289617. doi: 10.1371/journal.pone.0289617 (PMC10427022; doi:10.1371/journal.pone.0289617)
Supplement: S1 File — (DOCX) [file pone.0289617.s002.docx]

**Supporting information**

**S2: Developed countries where the prevalence of intrauterine fetal death is under 5 per 1000 live births**

| **Developed Country UN M59* 2021** | **Stillbirth rates 2019 UNICEF**** |
| --- | --- |
| Albania | 4.1 |
| Andorra | 2.1 |
| Australia | 2.2 |
| Austria | 2.2 |
| Belarus | 2.0 |
| Belgium | 2.8 |
| Bermuda | ND |
| Bosnia and Herzegovina | 2.8 |
| Canada | 2.8 |
| Channel Islands | ND |
| Chile | 3.1 |
| Croatia | 3.0 |
| Cyprus | 2.5 |
| Czechia | 2.6 |
| Denmark | 2.0 |
| Estonia | 2.2 |
| Faeroe Islands | ND |
| Finland | 2.0 |
| France | 4.3 |
| Germany | 2.7 |
| Greece | 3.1 |
| Hungary | 3.3 |
| Iceland | 1.9 |
| Ireland | 2.8 |
| Isle of Man | ND |
| Israel | 2.8 |
| Italy | 2.4 |
| Japan | 1.5 |
| Latvia | 3.2 |
| Liechtenstein | ND |
| Lithuania | 2.8 |
| Luxembourg | 3.4 |
| Malta | 3.0 |
| Monaco | 1.4 |
| Montenegro | 3.6 |
| Netherlands | 2.3 |
| New Zealand | 2.7 |
| Norway | 2.4 |
| Poland | 2.3 |
| Portugal | 2.5 |
| Romania | 3.2 |
| San Marino | 1.8 |
| Serbia | 4.4 |
| Singapore | 2.0 |
| Slovakia | 2.8 |
| Slovenia | 2.5 |
| Spain | 2.2 |
| Sweden | 2.4 |
| Switzerland | 2.2 |
| TFYR of Macedonia | ND |
| Ukraine | 4.5 |
| United Kingdom | 3.0 |
| USA | 3.0 |

Stillbirth rate (SBR): number of babies born with no sign of life at 28 weeks or more of gestation

ND = No SBR data available for 2019

The mortality rates used as underlying data and shown as “VR submitted to WHO/UNIGME 2020 version (VR)” are calculated by UNIGME using standard methods with data from Rosstat.

*According to the UN M49 standard for classification of economies by development status provided in the Classifications Newsletter, 2021 (link). Accessed 16 Nov 2021.

** According to UNICEF Data Warehouse, data from 2019. Accessed 16 Nov 2021.
