# Supplementary material for: Care and support when a baby is stillborn: A systematic review and an interpretive meta-synthesis of qualitative studies in high-income countries
Source: PLoS One. 2023 Aug 15;18(8):e0289617. doi: 10.1371/journal.pone.0289617 (PMC10427022; doi:10.1371/journal.pone.0289617)
Supplement: S2 File — (DOCX) [file pone.0289617.s003.docx]

# Supporting information

# S2: Search strategies for qualitative articles

### CINAHL via EBSCO 19 August 2022

Title: Perinatal death – experiences in parents, siblings and health personel

| Search terms | | Items found |
| --- | --- | --- |
| Population: Perinatal death | | |
|  | (MH "Perinatal Death") | 9,008 |
|  | TI (stillbirth* or "still birth*" or stillborn or "still born") OR AB (stillbirth* or "still birth*" or stillborn or "still born") OR SU (stillbirth* or "still birth*" or stillborn or "still born") | 5,898 |
|  | TI ( ((antenatal or "ante natal" or antepartal or "ante partal" or perinatal* or "perinatal" or peripartal or "peri partal" or fetal or foetal or intrauterine or "in utero") AND (bereave* or death or grief or griev* or demise or loss or mortality)) OR "pregnancy loss" ) OR AB ( ((antenatal or "ante natal" or antepartal or "ante partal" or perinatal* or "perinatal" or peripartal or "peri partal" or fetal or foetal or intrauterine or "in utero") N3 (bereave* or death or grief or griev* or demise or loss or mortality)) OR "pregnancy loss" ) OR SU ( ((antenatal or "ante natal" or antepartal or "ante partal" or perinatal* or "perinatal" or peripartal or "peri partal" or fetal or foetal or intrauterine or "in utero") N3 (bereave* or death or grief or griev* or demise or loss or mortality)) OR "pregnancy loss" ) | 16,010 |
|  | *1 OR 2 OR 3* | *18,315* |
| Evaluation: Experiences, expectations and attitudes in parents and siblings of stillborn children | | |
|  | (MH "Parents+/PF") OR (MH "Mothers+/PF") OR (MH "Fathers+/PF") OR (MH "Single Parent/PF") OR (MH "Parents of Disabled Children/PF") OR (MH "Siblings/PF") OR MH "Family Attitudes") OR (MH "Parental Attitudes") OR (MH "Maternal Attitudes") OR (MH "Paternal Attitudes) OR (MH "Attitude") OR (MH "Attitude to Health") OR (MH "Patient Satisfaction") OR (MH "Patient Preference") OR (MH "Personal Satisfaction") OR (MH "Attitude to Medical Treatment") OR (MH "Health Beliefs") OR (MH "Patient Attitudes") OR (MH "Consumer Satisfaction") OR (MH "Emotions") OR (MH "Patients/PF") | 303,994 |
|  | TI ((patient* or women or men or mother* or father* or brother* or sister* or sibling* or parent* or famil* or mum* or dad* or patern* or matern*) N15 (accept* or attitude* or belief* or believ* or disbelief* or disbeliev* or dissatif* or embodied or emotion* or encounter* or expectation* or experience* or improvement* or interpret* or involvement* or meaning or percept* or perspective* or psycholog* or satisf* or stigma* or relation or "self-report" or "treatment barrier*" or trust* or understanding* or unsatisf* or value or view*)) OR AB ((patient* or women or men or mother* or father* or brother* or sister* or sibling* or parent* or famil* or mum* or dad* or patern* or matern*) N15 (accept* or attitude* or belief* or believ* or disbelief* or disbeliev* or dissatif* or embodied or emotion* or encounter* or expectation* or experience* or improvement* or interpret* or involvement* or meaning or percept* or perspective* or psycholog* or satisf* or stigma* or relation or "self-report" or "treatment barrier*" or trust* or understanding* or unsatisf* or value or view*)) OR SU ((patient* or women or men or mother* or father* or brother* or sister* or sibling* or parent* or famil* or mum* or dad* or patern* or matern*) N15 (accept* or attitude* or belief* or believ* or disbelief* or disbeliev* or dissatif* or embodied or emotion* or encounter* or expectation* or experience* or improvement* or interpret* or involvement* or meaning or percept* or perspective* or psycholog* or satisf* or stigma* or relation or "self-report" or "treatment barrier*" or trust* or understanding* or unsatisf* or value or view*)) | 849,619 |
|  | *5 OR 6* | *954,332* |
| **Evaluation: Experiences, expectations and attitudes in health personnel** | | |
|  | (MH "Attitude of Health Personnel+") OR (MH "Health Personnel+/PF") | 155,696 |
|  | TI (("childbirth professional*" or counselor* or counsellor* or "general practitioner*" or gynaecologist* or "health car*" or "maternity ward*" or midwi* or nurs* or obstetrician* or physician* or professional* or psychotherapist* or staff) N15 (accept* or attitude* or belief* or believ* or disbelief* or disbeliev* or dissatif* or embodied or emotion* or encounter* or expectation* or experience* or improvement* or interpret* or involvement* or meaning or percept* or perspective* or psycholog* or satisf* or stigma* or relation or "self-report" or "treatment barrier*" or trust* or understanding* or unsatisf* or value or view*)) OR AB (("childbirth professional*" or counselor* or counsellor* or "general practitioner*" or gynaecologist* or "health car*" or "maternity ward*" or midwi* or nurs* or obstetrician* or physician* or professional* or psychotherapist* or staff) N15 (accept* or attitude* or belief* or believ* or disbelief* or disbeliev* or dissatif* or embodied or emotion* or encounter* or expectation* or experience* or improvement* or interpret* or involvement* or meaning or percept* or perspective* or psycholog* or satisf* or stigma* or relation or "self-report" or "treatment barrier*" or trust* or understanding* or unsatisf* or value or view*)) OR SU (("childbirth professional*" or counselor* or counsellor* or "general practitioner*" or gynaecologist* or "health car*" or "maternity ward*" or midwi* or nurs* or obstetrician* or physician* or professional* or psychotherapist* or staff) N15 (accept* or attitude* or belief* or believ* or disbelief* or disbeliev* or dissatif* or embodied or emotion* or encounter* or expectation* or experience* or improvement* or interpret* or involvement* or meaning or percept* or perspective* or psycholog* or satisf* or stigma* or relation or "self-report" or "treatment barrier*" or trust* or understanding* or unsatisf* or value or view*)) | 426,689 |
|  | *8 OR 9* | *489,159* |
| Study types: studies with qualitative methods | | |
|  | (MH "Qualitative Studies+") OR (MH "Empirical Research") OR (MH "Questionnaires+") OR (MH "Videorecording") OR (MH "Research, Nursing") OR (MH "Research, Midwifery") OR (MH "Survey Research") OR (MH "Phenomenological Research") OR (MH "Grounded Theory") OR (MH "Multimethod Studies") OR (MH "Content Analysis") OR (MH "Constant Comparative Method") OR (MH "Discourse Analysis") OR (MH "Thematic Analysis") OR (MH "Audiorecording") OR (MH "Focus Groups") OR (MH "Interviews+") OR (MH "Narratives+") OR (MH "Self Report+") OR (MH "Surveys+") OR (MH "Field Studies") OR (MH "Cluster Sample+") OR (MH "Comparative Studies") OR (MH "Life Experiences") OR (MH "Ethnology") OR (MH "Ethnonursing Research") OR (MH "Ethnological Research") OR (MH "Ethnographic Research") OR (MH "Phenomenology") OR (MH "Triangulation") OR (MH "Storytelling") | 1,384,592 |
|  | TI ( ("action research" or "cluster sample*" or "constant comparative method*" or "key informant*" or "maximum variation sampling*" or qualitative* or interview* or "focus group*" or phenomeno* or ethnolog* or ethnograph* or ethnonurs* or ethnomethodolog* or "meta-ethnograph*" or hermeneutic* or "grounded theory" or observation or "lived experience*" or narrat* or "mixed method*" or "content analys*" or "discourse analys*" or poststructur* or "purposive sample*" or "social systems theor*" or "thematic analys*" or "theoretical sample*" or "triangulation design") ) OR AB ( ("action research" or "cluster sample*" or "constant comparative method*" or "key informant*" or "maximum variation sampling*" or qualitative* or interview* or "focus group*" or phenomeno* or ethnolog* or ethnograph* or ethnonurs* or ethnomethodolog* or "meta-ethnograph*" or hermeneutic* or "grounded theory" or observation or "lived experience*" or narrat* or "mixed method*" or "content analys*" or "discourse analys*" or poststructur* or "purposive sample*" or "social systems theor*" or "thematic analys*" or "theoretical sample*" or "triangulation design") ) OR SU ( ("action research" or "cluster sample*" or "constant comparative method*" or "key informant*" or "maximum variation sampling*" or qualitative* or interview* or "focus group*" or phenomeno* or ethnolog* or ethnograph* or ethnonurs* or ethnomethodolog* or "meta-ethnograph*" or hermeneutic* or "grounded theory" or observation or "lived experience*" or narrat* or "mixed method*" or "content analys*" or "discourse analys*" or poststructur* or "purposive sample*" or "social systems theor*" or "thematic analys*" or "theoretical sample*" or "triangulation design") ) | 690,854 |
|  | TI ( ((field N2 (research or study or studies or work)) or (grounded N2 (theor* or study or studies or research or analys*)) or ((lived or life) N2 (experience* or story or stories)) or ((video or tape) N2 record*) or (("semi-structured" or semistructured or unstructured or informal or "in-depth" or indepth or "face-to-face" or structured or guide) N3 (interview* or discussion* or open ended or questionnaire*))) ) OR AB ( ((field N2 (research or study or studies or work)) or (grounded N2 (theor* or study or studies or research or analys*)) or ((lived or life) N2 (experience* or story or stories)) or ((video or tape) N2 record*) or (("semi-structured" or semistructured or unstructured or informal or "in-depth" or indepth or "face-to-face" or structured or guide) N3 (interview* or discussion* or open ended or questionnaire*))) ) OR SU ( ((field N2 (research or study or studies or work)) or (grounded N2 (theor* or study or studies or research or analys*)) or ((lived or life) N2 (experience* or story or stories)) or ((video or tape) N2 record*) or (("semi-structured" or semistructured or unstructured or informal or "in-depth" or indepth or "face-to-face" or structured or guide) N3 (interview* or discussion* or open ended or questionnaire*))) ) | 238,044 |
|  | TI ( (mixed or multi) W1 (method* or stud*) ) OR AB ( (mixed or multi) W1 (method* or stud*) ) OR SU ( (mixed or multi) W1 (method* or stud*) ) | 31,490 |
|  | *11 OR 12 OR 13 OR 14* | *1,645,000* |
| Combined sets: | | |
|  | 4 AND (7 OR 10 OR 15) | 7,441 |
| Final result | | |
|  | 16 Limiters - Language: Danish, English, Norwegian, Swedish | 7,298 |

The final search result, usually found at the end of the documentation, forms the list of abstracts.

**AB** = Abstract; **AU** = Author; **DE** = Term from the thesaurus; **MH**= Exact Subject Heading from CINAHL Subject Headings; **MM** = Major Concept; **TI** = Title; **TX** = All Text. Performs a keyword search of all the database's searchable fields; **ZC** = Methodology Index; ***** = Truncation; **“ “** = Citation Marks; searches for an exact phrase; **N** = Near Operator (N) finds the words if they are a maximum of x words apart from one another, regardless of the order in which they appear.; **W** = Within Operator (W) finds the words if they are within x words of one another, in the order in which you entered them.

### Cochrane Library via Wiley 19 August 2022 (CENTRAL)

Title: Perinatal death – experiences in parents, siblings and health personel

| Search terms | | Items found |
| --- | --- | --- |
| Population: Perinatal death | | |
|  | MeSH descriptor: [Stillbirth] explode all trees | 151 |
|  | MeSH descriptor: [Fetal Death] explode all trees | 394 |
|  | (Stillbirth OR "still birth" OR stillborn OR "still born"):ti, ab, kw | 1348 |
|  | (((antenatal or "ante natal" or antepartal or "ante partal" or perinatal* or "peri natal" or peripartal or "peri partal" or fetal or foetal or intrauterine or "in utero") NEAR1 (bereave* or death or grief or griev* or demise or loss or mortality))):ti,ab,kw OR ("pregnancy loss"):ti,ab,kw | 885 |
| Final result | | |
|  | **1 OR 2 OR 3 OR 4** | **CENTRAL/**  **1794** |

The final search result, usually found at the end of the documentation, forms the list of abstracts.

**:au** = Author; MeSH = Term from the Medline controlled vocabulary, including terms found below this term in the MeSH hierarchy; **this term only** = Does not include terms found below this term in the MeSH hierarchy; **:ti** = Title; **:ab** = Abstract; **:kw** = Keyword; ***** = Truncation; **“ “** = Citation Marks; searches for an exact phrase; **CDSR** = Cochrane Database of Systematic Review; **Cochrane Protocols** = Protocols of systematic reviews registered in Cochrane Library; **CENTRAL** = Cochrane Central Register of Controlled Trials, “trials”

### Embase via Elsevier 19 August 2022

Title: Perinatal death – experiences in parents, siblings and health personel

| Search terms | | Items found |
| --- | --- | --- |
| Population: Perinatal death | | |
|  | 'stillbirth'/mj OR 'fetus death'/mj | 13,940 |
|  | stillbirth:ti OR 'still birth':ti OR stillborn:ti OR 'still born':ti | 3,528 |
|  | (antenatal:ti OR 'ante natal':ti OR antepartal:ti OR 'ante partal':ti OR perinatal*:ti OR 'perinatal':ti OR peripartal:ti OR 'peri partal':ti OR fetal:ti OR foetal:ti OR intrauterine:ti OR 'in utero':ti) AND (bereave*:ti OR death:ti OR grief:ti OR griev*:ti OR demise:ti OR loss:ti OR mortality:ti) OR 'pregnancy loss':ti | 14,384 |
|  | *1 OR 2 OR 3* | *25,019* |
| Evaluation: Experiences, expectations and attitudes in parents and siblings of stillborn children **Evaluation: Experiences, expectations and attitudes in health personnel** | | |
|  | 'patient attitude' OR 'patient preference'/exp/mj OR 'patient satisfaction'/de OR 'family attitude'/exp OR 'health personnel attitude'/exp | 473,012 |
|  | (patient*:ti OR women:ti OR men:ti OR mother*:ti OR father*:ti OR brother*:ti OR sister*:ti OR sibling*:ti OR parent*:ti OR famil*:ti OR mum*:ti OR dad*:ti OR patern*:ti OR matern*:ti) AND (accept*:ti OR attitude*:ti OR belief*:ti OR believ*:ti OR disbelief*:ti OR disbeliev*:ti OR dissatif*:ti OR embodied:ti OR emotion*:ti OR encounter*:ti OR expectation*:ti OR experience*:ti OR improvement*:ti OR interpret*:ti OR involvement*:ti OR meaning:ti OR percept*:ti OR perspective*:ti OR psycholog*:ti OR satisf*:ti OR stigma*:ti OR relation:ti OR 'self-report':ti OR 'treatment barrier*':ti OR trust*:ti OR understanding*:ti OR unsatisf*:ti OR value:ti OR view*:ti) | 319,370 |
|  | ('childbirth professional*':ti OR counselor*:ti OR counsellor*:ti OR 'general practitioner*':ti OR gynaecologist*:ti OR 'health car*':ti OR 'maternity ward*':ti OR midwi*:ti OR nurs*:ti OR obstetrician*:ti OR physician*:ti OR professional*:ti OR psychotherapist*:ti OR staff:ti) AND (accept*:ti OR attitude*:ti OR belief*:ti OR believ*:ti OR disbelief*:ti OR disbeliev*:ti OR dissatif*:ti OR embodied:ti OR emotion*:ti OR encounter*:ti OR expectation*:ti OR experience*:ti OR improvement*:ti OR interpret*:ti OR involvement*:ti OR meaning:ti OR percept*:ti OR perspective*:ti OR psycholog*:ti OR satisf*:ti OR stigma*:ti OR relation:ti OR 'self-report':ti OR 'treatment barrier*':ti OR trust*:ti OR understanding*:ti OR unsatisf*:ti OR value:ti OR view*:ti) | 96,072 |
|  | *5 OR 6 OR 7* | *795,820* |
| Study types: studies with qualitative method | | |
|  | 'qualitative research'/exp OR 'empirical research'/exp OR 'questionnaire'/exp OR 'nursing research'/exp OR 'phenomenology'/exp OR 'survey methodology'/exp OR 'grounded theory'/exp OR 'content analysis'/exp OR 'multimethod study'/exp OR 'constant comparative method'/exp OR 'discourse analysis'/exp OR 'thematic analysis'/exp OR 'self report'/exp OR 'narrative'/exp OR 'interview'/exp OR 'comparative study'/de OR 'field study'/exp OR 'ethnology'/exp OR 'cluster analysis'/exp OR 'storytelling'/exp OR 'personal experience'/exp OR 'recording'/exp | 2,591,482 |
|  | 'action research' OR 'cluster sample*' OR 'constant comparative method*' OR 'key informant*' OR 'maximum variation sampling*' OR qualitative* OR interview* OR 'focus group*' OR phenomeno* OR ethnolog* OR ethnograph* OR ethnonurs* OR ethnomethodolog* OR 'meta-ethnograph*' OR hermeneutic* OR 'grounded theory' OR observation OR 'lived experience*' OR narrat* OR 'mixed method*' OR 'content analys*' OR 'discourse analys*' OR poststructur* OR 'purposive sample*' OR 'social systems theor*' OR 'thematic analys*' OR 'theoretical sample*' OR 'triangulation design' | 1,660,948 |
|  | (field NEAR/2 (research OR study OR studies OR work)):ti | 46,946 |
|  | (('semi-structured' OR semistructured OR unstructured OR informal OR 'in-depth' OR indepth OR 'face-to-face' OR structured OR guide) NEAR/3 (interview* OR discussion* OR 'open ended' OR questionnaire*)):ti | 205,082 |
|  | ((video OR tape) NEAR/2 record*):ti | 22,120 |
|  | ((lived OR life) NEAR/2 (experience* OR story OR stories)):ti | 6,144 |
|  | (grounded NEAR/2 (theor* OR study OR studies OR research OR analys*)):ti | 18,829 |
|  | *9 OR 10 OR 11 OR 12 OR 13 OR 14 OR 15* | *3,787,795* |
| Limits: language, publication type | | |
|  | ([danish]/lim OR [english]/lim OR [norwegian]/lim OR [swedish]/lim) |  |
|  | [preprint]/lim |  |
| Combined sets | | |
|  | *4 AND (8 OR 16)* | *2,662* |
| Final result | | |
|  | (17 AND 19) NOT 18 | 2,383 |

The final search result, usually found at the end of the documentation, forms the list of abstracts.

**/de** = Term from the EMTREE controlled vocabulary; **/exp** = Includes terms found below this term in the EMTREE hierarchy

**/mj** = Major Topic; **:ab** = Abstract; **:au** = Author; **:ti** = Article Title; **:ti,ab** = Title or abstract; ***** = Truncation; **' '** = Citation Marks; searches for an exact phrase; **NEAR/n** = Requests terms that are within 'n' words of each other in either direction; **NEXT/n** = Requests terms that are within 'n' words of each other in the order specified

### Medline via OvidSP 19 August 2022

Title: Perinatal death – experiences in parents, siblings and health personel

| Search terms | | Items found |
| --- | --- | --- |
| Population: Perinatal death | | |
|  | fetal death/ or stillbirth/ | 29847 |
|  | (stillbirth* or "still birth*" or stillborn or "still born").ab,ti. | 18142 |
|  | (((antenatal or "ante natal" or antepartal or "ante partal" or perinatal* or "peri natal" or peripartal or "peri partal" or fetal or foetal or intrauterine or "in utero") adj3 (bereave* or death or grief or griev* or demise or loss or mortality)) or "pregnancy loss").ab,ti. | 37357 |
|  | *1 OR 2 OR 3* | *65840* |
| Evaluation: Experiences, expectations and attitudes in parents and siblings of stillborn children | | |
|  | exp Family/px [Psychology] | 101249 |
|  | Siblings/px [Psychology] | 1653 |
|  | Parents/px [Psychology] | 31482 |
|  | attitude to health/ or "patient acceptance of health care"/ or patient satisfaction/ or patient preference/ | 228079 |
|  | Emotions/ | 79432 |
|  | Expressed Emotion/ | 1835 |
|  | exp Patients/px [Psychology] | 18406 |
|  | ((patient* or women or men or mother* or father* or brother* or sister* or sibling* or parent* or famil* or mum* or dad* or patern* or matern*) adj15 (accept* or attitude* or belief* or believ* or disbelief* or disbeliev* or dissatif* or embodied or emotion* or encounter* or expectation* or experience* or improvement* or interpret* or involvement* or meaning or percept* or perspective* or psycholog* or satisf* or stigma* or relation or "self-report" or "treatment barrier*" or trust* or understanding* or unsatisf* or value or view*)).ti,kf. | 1686763 |
|  | *5 OR 6 OR 7 OR 8 OR 9 OR 10 OR 11 OR 12* | *1927682* |
| **Evaluation: Experiences, expectations and attitudes in health personnel** | | |
|  | exp Health Personnel/px [Psychology] | 124551 |
|  | attitude/ or "attitude of health personnel"/ | 181210 |
|  | (("childbirth professional*" or counselor* or counsellor* or "general practitioner*" or gynaecologist* or "health car*" or "maternity ward*" or midwi* or nurs* or obstetrician* or physician* or professional* or psychotherapist* or staff) adj15 (accept* or attitude* or belief* or believ* or disbelief* or disbeliev* or dissatif* or embodied or emotion* or encounter* or expectation* or experience* or improvement* or interpret* or involvement* or meaning or percept* or perspective* or psycholog* or satisf* or stigma* or relation or "self-report" or "treatment barrier*" or trust* or understanding* or unsatisf* or value or view*)).ti,kf. | 378127 |
|  | *14 OR 15 OR 16* | *572592* |
| Study types: studies with qualitative method | | |
|  | exp empirical research/ or exp qualitative research/ | 81403 |
|  | data collection/ or focus groups/ or interviews as topic/ or narration/ or "surveys and questionnaires"/ or health care surveys/ or patient reported outcome measures/ or health surveys/ or patient health questionnaire/ or self report/ | 825230 |
|  | Ethnology/ | 1592 |
|  | exp Nursing Research/ | 53394 |
|  | exp comparative study/ | 1911467 |
|  | personal narratives as topic/ | 353 |
|  | personal narrative/ | 6198 |
|  | exp Video Recording/ | 44166 |
|  | ("action research" or "cluster sample*" or "constant comparative method*" or "key informant*" or "maximum variation sampling*" or qualitative* or interview* or "focus group*" or phenomeno* or ethnolog* or ethnograph* or ethnonurs* or ethnomethodolog* or "meta-ethnograph*" or hermeneutic* or "grounded theory" or observation or "lived experience*" or narrat* or "mixed method*" or "content analys*" or "discourse analys*" or poststructur* or "purposive sample*" or "social systems theor*" or "thematic analys*" or "theoretical sample*" or "triangulation design").ab,kf,ti. | 1332965 |
|  | ((field adj3 (research or study or studies or work)) or (grounded adj2 (theor* or study or studies or research or analys*)) or ((lived or life) adj2 (experience* or story or stories)) or ((video or tape) adj2 record*) or (("semi-structured" or semistructured or unstructured or informal or "in-depth" or indepth or "face-to-face" or structured or guide) adj3 (interview* or discussion* or open ended or questionnaire*))).ab,kf,ti. | 273263 |
|  | *18 OR 19 OR 20 OR 21 OR 22 OR 23 OR 24 OR 25 OR 26 OR 27* | *3907067* |
| Limits: human studies | | |
|  | exp Animals/ NOT exp Humans/ | 5038073 |
| Combined sets | | |
|  | *4 AND (13 OR 17 OR 28)* | *9640* |
| Final result | | |
|  | 30 NOT 29 **limit to (danish or english or norwegian or swedish)** | 7834 |

The final search result, usually found at the end of the documentation, forms the list of abstracts.

**.ab.** = Abstract; .**ab,ti.** = Abstract or title; **.af.** = All fields; **Exp** = Term from the Medline controlled vocabulary, including terms found below this term in the MeSH hierarchy; **.sh.** = Term from the Medline controlled vocabulary; **.ti.** = Title; **/** = Term from the Medline controlled vocabulary, but does not include terms found below this term in the MeSH hierarchy; ***** = Focus (if found in front of a MeSH-term); *** or $** = Truncation (if found at the end of a free text term); **.mp** = Text, heading word, subject area node, title; **“ “** = Citation Marks; searches for an exact phrase; *ADJn* = Positional operator that lets you retrieve records that contain your terms (in any order) within a specified number (n) of words of each other.

### PsycINFO via EBSCO 19 August 2022

Title: Perinatal death – experiences in parents, siblings and health personel

| Search terms | | Items found |
| --- | --- | --- |
| Population: Perinatal death | | |
|  | (DE "Death and Dying") AND (DE "Pregnancy Outcomes" OR DE "Birth" OR DE "Induced Abortion" OR DE "Obstetrical Complications" OR DE "Spontaneous Abortion") | 539 |
|  | TI (stillbirth* or "still birth*" or stillborn or "still born") OR AB (stillbirth* or "still birth*" or stillborn or "still born") OR SU (stillbirth* or "still birth*" or stillborn or "still born") | 1,068 |
|  | TI ( ((antenatal or "ante natal" or antepartal or "ante partal" or perinatal* or "peri natal" or peripartal or "peri partal" or fetal or foetal or intrauterine or "in utero") N1 (bereave* or death or grief or griev* or demise or loss or mortality)) OR "pregnancy loss" ) OR AB ( ((antenatal or "ante natal" or antepartal or "ante partal" or perinatal* or "perinatal" or peripartal or "peri partal" or fetal or foetal or intrauterine or "in utero") N1 (bereave* or death or grief or griev* or demise or loss or mortality)) OR "pregnancy loss" ) OR SU ( ((antenatal or "ante natal" or antepartal or "ante partal" or perinatal* or "perinatal" or peripartal or "peri partal" or fetal or foetal or intrauterine or "in utero") N1 (bereave* or death or grief or griev* or demise or loss or mortality)) OR "pregnancy loss" ) | 1,834 |
|  | *1 OR 2 OR 3* | *2,825* |
| Final result | | |
|  | 4 Limiters - Language: Danish, English, Norwegian, Swedish | 2,711 |

The final search result, usually found at the end of the documentation, forms the list of abstracts.

**AB** = Abstract; **AU** = Author; **DE** = Term from the thesaurus; **MH**= Exact Subject Heading from CINAHL Subject Headings; **MM** = Major Concept; **TI** = Title; **TX** = All Text. Performs a keyword search of all the database's searchable fields; **ZC** = Methodology Index; ***** = Truncation; **“ “** = Citation Marks; searches for an exact phrase; **N** = Near Operator (N) finds the words if they are a maximum of x words apart from one another, regardless of the order in which they appear.; **W** = Within Operator (W) finds the words if they are within x words of one another, in the order in which you entered them.

### Scopus via Elsevier 11 March 2021

Title: Perinatal death – citation search

| Search terms | | Items found |
| --- | --- | --- |
| Cited articles | | |
|  | ( DOI ( "10.1007/s00404-017-4309-9" OR "10.1016/j.ejogrb.2018.01.007" OR "10.1016/j.ijnurstu.2012.10.014" OR "10.1016/j.jogn.2015.10.010" OR "10.1016/j.jogn.2020.09.156" OR "10.1016/j.midw.2013.10.021" OR "10.1016/j.midw.2018.06.011" OR "10.1016/j.midw.2018.08.010" OR "10.1016/j.midw.2019.06.013" OR "10.1016/j.wombi.2019.05.013" OR "10.1046/j.1523-536x.2001.00124.x" ) ) OR ( DOI ( "10.1080/02646838.2012.661849" OR "10.1080/02682621.2020.1828724" OR "10.1080/07481187.2013.809034" OR "10.1080/07481187.2019.1626936" OR "10.1097/ANC.0000000000000703" OR "10.1097/NMC.0000000000000490" OR "10.1111/1471-0528.12695" OR "10.1111/1471-0528.16113" OR "10.1111/1552-6909.12040" OR "10.1111/ajo.12684" OR "10.1111/aogs.13354" OR "10.1111/birt.12335" OR "10.1111/j.0730-7659.2004.00291.x" ) ) OR ( DOI ( "10.1111/j.0730-7659.2004.00304.x" OR "10.1111/j.1523-536X.2010.00457.x" OR "10.1111/j.1552-6909.1997.tb01506.x" OR "10.1111/scs.12816" OR "10.1136/bmjopen-2012-002237" OR "10.1186/1471-2393-12-137" OR "10.1186/s12884-020-02865-4" OR "10.1371/journal.pone.0191635" OR "10.2190/om.57.1.a" OR "10.12707/RV20037" OR "10.12968/bjom.2016.24.10.715" OR "10.20467/1091-5710.14.4.14" OR "10.1016/j.srhc.2011.02.002" OR "10.1177/00302228211050500" OR "10.1186/1471-2393-14-203" ) ) OR ( PMID ( 24968620 OR 17964235 ) ) OR ( ( TITLE ( "helping a woman" ) AND AUTHOR-NAME ( radestad ) ) ) | 40 |
|  | **View cited by** | **565** |

The final search result, usually found at the end of the documentation, forms the list of abstracts.

**TITLE-ABS-KEY** = Title or abstract or keywords; **ALL =** All fields; **PRE/n =** "precedes by". The first term in the search must precede the second by a specified number of terms (n).; **W/n =** "Within". The terms in the search must be within a specified number of terms (n) in any order.; ***** = Truncation; **“ “** = Citation Marks; searches for an exact phrase; **LIMIT-TO (SRCTYPE, "j"** = Limit to source type journal; **LIMIT-TO (DOCTYPE, "ar"** = Limit to document type article; **LIMIT-TO (DOCTYPE, "re"** = Limit to document type review

### Cited articles

1. Agwu Kalu F, Coughlan B, Larkin P. A mixed methods sequential explanatory study of the psychosocial factors that impact on midwives’ confidence to provide bereavement support to parents who have experienced a perinatal loss. Midwifery. 2018;64:69-76.

2. Alghamdi R, Jarrett P. Experiences of student midwives in the care of women with perinatal loss: A qualitative descriptive study. British Journal of Midwifery. 2016;24(10):715-22.

3. Avelin P, Erlandsson K, Hildingsson I, Rådestad I. Swedish parents' experiences of parenthood and the need for support to siblings when a baby is stillborn. Birth. 2011;38(2):150-8.

4. Avelin P, Gyllenswärd G, Erlandsson K, Rådestad I. Adolescents' Experiences of Having a Stillborn Half-Sibling. Death Studies. 2014;38(9):557-62.

5. Bond D, Raynes-Greenow C, Gordon A. Bereaved parents’ experience of care and follow-up after stillbirth in Sydney hospitals. Australian and New Zealand Journal of Obstetrics and Gynaecology. 2018;58(2):185-91.

6. Brierley-Jones L, Crawley R, Jones E, Gordon I, Knight J, Hinshaw K. Supporting parents through stillbirth: A qualitative study exploring the views of health professionals and health care staff in three hospitals in England. European Journal of Obstetrics and Gynecology and Reproductive Biology. 2018;222:45-51.

7. Cacciatore J, Erlandsson K, Rådestad I. Fatherhood and suffering: A qualitative exploration of Swedish men's experiences of care after the death of a baby. International Journal of Nursing Studies. 2013;50(5):664-70.

8. Camacho Ávila M, Fernández Medina IM, Jiménez-López FR, Granero-Molina J, Hernández-Padilla JM, Hernández Sánchez E, et al. Parents' Experiences about Support Following Stillbirth and Neonatal Death. Adv Neonat Care. 2020;20(2):151-60.

9. Cassidy PR. The Disenfranchisement of Perinatal Grief: How Silence, Silencing and Self-Censorship Complicate Bereavement (a Mixed Methods Study). Omega (United States). 2021.

10. Downe S, Schmidt E, Kingdon C, Heazell AEP. Bereaved parents' experience of stillbirth in UK hospitals: A qualitative interview study. BMJ Open. 2013;3(2).

11. Fernández-Alcántara M, Schul-Martin L, García Caro MP, Montoya-Juárez R, Pérez-Marfil MN, Zech E. ‘In the hospital there are no care guidelines’: experiences and practices in perinatal loss in Spain. Scandinavian Journal of Caring Sciences. 2020;34(4):1063-73.

12. Gillis C, Wheatley V, Jones A, Roland B, Gill M, Marlett N, et al. Stillbirth, still life: A qualitative patient-led study on parents’ unsilenced stories of stillbirth. Bereavement Care. 2020;39(3):124-32.

13. Hutti MH, Polivka B, White S, Hill J, Clark P, Cooke C, et al. Experiences of Nurses Who Care for Women After Fetal Loss. JOGNN - Journal of Obstetric, Gynecologic, and Neonatal Nursing. 2016;45(1):17-27.

14. Kavanaugh K. Parents' experience surrounding the death of a newborn whose birth is at the margin of viability. Journal of obstetric, gynecologic, and neonatal nursing : JOGNN / NAACOG. 1997;26(1):43-51.

15. Kelley MC, Trinidad SB. Silent loss and the clinical encounter: Parents' and physicians' experiences of stillbirth-a qualitative analysis. BMC Pregnancy and Childbirth. 2012;12.

16. King MQ, Oka M, Robinson WD. Pain without reward: A phenomenological exploration of stillbirth for couples and their hospital encounter. Death Studies. 2021;45(2):152-62.

17. Lee C. 'She was a person, she was here': The experience of late pregnancy loss in Australia. Journal of Reproductive and Infant Psychology. 2012;30(1):62-76.

18. Listermar KH, Sormunen T, Rådestad I. Perinatal palliative care after a stillbirth—Midwives’ experiences of using Cubitus baby. Women and Birth. 2020;33(2):161-4.

19. Malm MC, Rådestad I, Erlandsson K, Lindgren H. Waiting in no-man's-land - Mothers' experiences before the induction of labour after their baby has died in utero. Sexual and Reproductive Healthcare. 2011;2(2):51-5.

20. Martínez-Serrano P, Palmar-Santos AM, Solís-Muñoz M, Álvarez-Plaza C, Pedraz-Marcos A. Midwives’ experience of delivery care in late foetal death: A qualitative study. Midwifery. 2018;66:127-33.

21. Martínez-Serrano P, Pedraz-Marcos A, Solís-Muñoz M, Palmar-Santos AM. The experience of mothers and fathers in cases of stillbirth in Spain. A qualitative study. Midwifery. 2019;77:37-44.

22. McCreight BS. Perinatal loss: A qualitative study in Northern Ireland. Omega: Journal of Death and Dying. 2008;57(1):1-19.

23. McNamara K, Meaney S, O'Donoghue K. Intrapartum fetal death and doctors: a qualitative exploration. Acta Obstetricia et Gynecologica Scandinavica. 2018;97(7):890-8.

24. McNamara K, Meaney S, O’Connell O, McCarthy M, Greene RA, O’Donoghue K. Healthcare professionals’ response to intrapartum death: a cross-sectional study. Archives of Gynecology and Obstetrics. 2017;295(4):845-52.

25. Miranda AMC, Zangão MOB. Mothers’ experiences of fetal death. Revista de Enfermagem Referencia. 2020;2020(3):1-8.

26. Nurse-Clarke N. Managing Ambiguity When Caring for Women Who Experience Stillbirth. JOGNN - Journal of Obstetric, Gynecologic, and Neonatal Nursing. 2021;50(2):143-53.

27. Nuzum D, Meaney S, O'Donoghue K. The impact of stillbirth on consultant obstetrician gynaecologists: A qualitative study. BJOG: An International Journal of Obstetrics and Gynaecology. 2014;121(8):1020-8.

28. Nuzum D, Meaney S, O’Donoghue K. The impact of stillbirth on bereaved parents: A qualitative study. PLoS ONE. 2018;13(1).

29. Puia DM, Lewis L, Beck CT. Experiences of obstetric nurses who are present for a perinatal loss. JOGNN - Journal of Obstetric, Gynecologic, and Neonatal Nursing. 2013;42(3):321-31.

30. Redshaw M, Henderson J. Care associated with stillbirth for the most disadvantaged women: A multi-method study of care in England. Birth. 2018;45(3):275-85.

31. Ryninks K, Roberts-Collins C, McKenzie-McHarg K, Horsch A. Mothers' experience of their contact with their stillborn infant: An interpretative phenomenological analysis. BMC Pregnancy and Childbirth. 2014;14(1).

32. Rådestad I, Christoffersen L. Helping a woman meet her stillborn baby while it is soft and warm. British Journal of Midwifery. 2008;16(9):588-91.

33. Rådestad I, Malm MC, Lindgren H, Pettersson K, Larsson LLF. Being alone in silence - Mothers' experiences upon confirmation of their baby's death in utero. Midwifery. 2014;30(3):e91-e5.

34. Samuelsson M, Rådestad I, Segesten K. A waste of life: Fathers' experience of losing a child before birth. Birth. 2001;28(2):124-30.

35. Skupski D. We need bereavement training and support for stillbirth providers. BJOG: An International Journal of Obstetrics and Gynaecology. 2014;121(8):1028.

36. Smith LK, Dickens J, Bender Atik R, Bevan C, Fisher J, Hinton L. Parents’ experiences of care following the loss of a baby at the margins between miscarriage, stillbirth and neonatal death: a UK qualitative study. BJOG: An International Journal of Obstetrics and Gynaecology. 2020;127(7):868-74.

37. Smith P, Vasileiou K, Jordan A. Healthcare professionals' perceptions and experiences of using a cold cot following the loss of a baby: A qualitative study in maternity and neonatal units in the UK. BMC Pregnancy and Childbirth. 2020;20(1).

38. Säflund K, Sjögren B, Wredling R. The role of caregivers after a stillbirth: Views and experiences of parents. Birth. 2004;31(2):132-7.

39. Trulsson O, Rådestad I. The silent child - Mothers' experiences before, during, and after stillbirth. Birth. 2004;31(3):189-95.

40. Willis P. Nurses' Perspective on Caring for Women Experiencing Perinatal Loss. MCN The American Journal of Maternal/Child Nursing. 2019;44(1):46-51.
