# Supplementary material for: Care and support when a baby is stillborn: A systematic review and an interpretive meta-synthesis of qualitative studies in high-income countries
Source: PLoS One. 2023 Aug 15;18(8):e0289617. doi: 10.1371/journal.pone.0289617 (PMC10427022; doi:10.1371/journal.pone.0289617)
Supplement: S3 File — (DOCX) [file pone.0289617.s004.docx]

Supporting information

S4: Excluded studies

**Table of Contents**

[Qualitative studies excluded by relevance 1](#_bookmark0)

[Qualitative studies excluded due to methodological limitations 32](#_bookmark1)

Qualitative studies excluded by relevance

| Reference | Reason for exclusion |
| --- | --- |
| Aagaard H, Storm I, Klitgaard J. Losing one twin in the NICU – A case study of the parental experience. Journal of Neonatal Nursing, 2016; 22  (4): 153-58. | Population not relevant |
| Abdollahpour S, Khosravi A, Bolbolhaghighi N. The effect of the magical hour on post-traumatic stress disorder (PTSD) in traumatic childbirth: a clinical trial. Journal of Reproductive and Infant Psychology, 2016; 34 (4): 403-12. | Setting not relevant |
| Alexander KV. 'The one thing you can never take away': perinatal bereavement photographs. MCN: The American Journal of Maternal Child Nursing, 2001; 26 (3): 123-61. | Study design not relevant |
| Alghamdi R, Jarrett P. Experiences of student midwives in the care of women with perinatal loss: A qualitative descriptive study. British Journal of Midwifery, 2016; 24 (10): 715-22. | Perspective not relevant |
| Allahdadian M, Irajpour A, Kazemi A, Kheirabadi G. Social support: An approach to maintaining the health of women who have experienced stillbirth. Iranian Journal of Nursing and Midwifery Research, 2015; 20 (4): 465-70. | Setting not relevant |
| André B, Dahlø R, Eilertsen T, Hildingsson I, Shorey S, Ringdal GI. Coping Strategies of Norwegian Healthcare Professionals Facing | Outcomes not relevant |

**SBU** Statens beredning för medicinsk och social utvärdering • [www.sbu.se](http://www.sbu.se/)

| Reference | Reason for exclusion |
| --- | --- |
| Perinatal Death—A Qualitative Study. International Journal of Childbirth, 2019; 9 (3): 107-19. |  |
| Andrus M. Exhibition and Film About Miscarriage, Infertility, and Stillbirth: Art Therapy Implications. Art Therapy: Journal of the American Art Therapy Association, 2020; 37 (4): 169-76. | Outcomes not relevant |
| Attia L, Nolan A. Caring for parents following the death of a twin: A student's experience. British Journal of Midwifery, 2011; 19 (10): 665-  69. | Study design not relevant |
| Avelin P, Erlandsson K, Hildingsson I, Davidsson Bremborg A, Rådestad I. Make the Stillborn Baby and the Loss Real for the Siblings: Parents' Advice on How the Siblings of a Stillborn Baby Can Be Supported. Journal of Perinatal Education, 2012; 21 (2): 90-98. | Outcomes not relevant |
| Avelin P, Erlandsson K, Hildingsson I, Rådestad I. Swedish Parents' Experiences of Parenthood and the Need for Support to Siblings When a Baby is Stillborn. Birth: Issues in Perinatal Care, 2011; 38 (2): 150-58. | Outcomes not relevant |
| Avelin P, Radestad I, Saflund K, Wredling R, Erlandsson K. Parental grief and relationships after the loss of a stillborn baby. Midwifery, 2013; 29 (6): 668-73. | Outcomes not relevant |
| Aydin R, Körükcü Ö, Kabukcuoğlu K. Investigation of the Experiences of Mothers Living Through Prenatal Loss Incidents: A Qualitative Study. Journal of Nursing Research (Lippincott Williams & Wilkins), 2019; 27 (3): e22-e22. | Population not relevant |
| Bakhbakhi D, Burden C, Siassakos D, Hinton L, Duffy J, Flenady V, et al. Development of a core outcome set and identification of outcome measurement tools to improve care and research after stillbirth-the iCHOOSE Study. BJOG: An International Journal of Obstetrics and Gynaecology, 2019; 126216. | Study design not relevant |
| Bakhbakhi D, Burden C, Storey C, Heazell AE, Lynch M, Timlin L, et al. PARENTS 2 Study: a qualitative study of the views of healthcare professionals and stakeholders on parental engagement in the perinatal mortality review-from 'bottom of the pile' to joint learning. BMJ Open, 2019; 8 (11): e023792. | Outcomes not relevant |
| Bakhbakhi D, Khan K, Duffy J, Fraser A, Brookes S, Siassakos D. Exploring parental involvement in core outcome set development following a stillbirth, part of the International Initiative: International Collaboration for Harmonising Outcomes for Stillbirth (iCHOOSE). Journal of Evidence-Based Medicine, 2017; 1035-36. | Study design not relevant |

| Reference | Reason for exclusion |
| --- | --- |
| Bakhbakhi D, Siassakos D, Burden C, Jones F, Yoward F, Redshaw M, et al. Learning from deaths: Parents' Active Role and ENgagement in The review of their Stillbirth/perinatal death (the PARENTS 1 study). BMC Pregnancy and Childbirth, 2017; 17 (1). | Outcomes not relevant |
| Bakker JK, Paris J. Bereavement and religion online: Stillbirth, neonatal loss, and parental religiosity. Journal for the Scientific Study of Religion, 2013; 52 (4): 657-74. | Outcomes not relevant |
| Barry M, Quinn C, Bradshaw C, Noonan M, Brett M, Atkinson S, et al. Exploring perinatal death with midwifery students' using a collaborative art project. Nurse Education Today, 2017; 481-6. | Outcomes not relevant |
| Beaudoin MA, Ouellet N. [An exploration of factors influencing nursing practice with families experiencing perinatal loss ]. Rech Soins Infirm, 2018; (133): 58-69. | Language not relevant |
| Beck E, Gibson N, Heazell A. 'Real experiences which increase empathy' – a preliminary exploration of the utility of an audio archive describing parents' and clinicians' experiences of stillbirth. Bereavement Care, 2019; 38 (1): 33-41. | Study design not relevant |
| Bennett SM. Development and preliminary evaluation of a cognitive behavioral intervention for perinatal grief. Vol. 70, ProQuest Information & Learning, 2010, pp. 4475-75. | Study design not relevant |
| Blackwell L, Heyes J. Environmental Impacts of the Labor Ward on Bereaved Families. Journal of Prenatal & Perinatal Psychology & Health, 2022; 36 (1): 96-112. | Outcomes not relevant |
| Blood C, Cacciatore J. Best practice in bereavement photography after perinatal death: qualitative analysis with 104 parents. BMC Psychol, 2014; 2 (1): 15. | Population not relevant |
| Blood C, Cacciatore J. Parental grief and memento mori photography: narrative, meaning, culture, and context. Death Stud, 2014; 38 (1-5): 224-33. | Study design not relevant |
| Bond DM, Raynes-Greenow CH, Gordon A. Hospital care and follow- up after stillbirth: Lessons from parents. Journal of Paediatrics and Child Health, 2014; 5071. | Study design not relevant |
| Bonnette S, Broom A. On grief, fathering and the male role in men’s accounts of stillbirth. Journal of Sociology, 2012; 48 (3): 248-65. | Outcomes not relevant |

| Reference | Reason for exclusion |
| --- | --- |
| Bornemisza AY, Javor R, Erdos MB. Sibling Grief over Perinatal Loss—A Retrospective Qualitative Study. Journal of Loss and Trauma, 2021; 27 (6): 530-46. | Outcomes not relevant |
| Boyle FM, Horey D, Dean JH, Lohan A, Middleton P, Flenady V. Perinatal bereavement care during COVID-19 in Australian maternity settings. J Perinat Med, 2022; 50 (6): 822-31. | Outcomes not relevant |
| Boyle FM, Horey D, Wilson P, Ibiebele I, Schirmann A, Ellwood D, et al. Autopsy decision-making after stillbirth: Parents' perspectives.  Journal of Paediatrics and Child Health, 2016; 5216-17. | Study design not relevant |
| Boyle FM, Mutch AJ, Barber EA, Carroll C, Dean JH. Supporting parents following pregnancy loss: a cross-sectional study of telephone peer supporters. BMC Pregnancy Childbirth, 2015; 15 (1): 291. | Outcomes not relevant |
| Brierley-Jones L, Crawley R, Gordon I, Hinshaw K, Jones E. Supporting parents through stillbirth: A qualitative, exploratory study involving a range of health care professionals and support staff. BJOG: An International Journal of Obstetrics and Gynaecology, 2016; 12391. | Study design not relevant |
| Brierley-Jones L, Crawley R, Lomax S, Ayers S. Stillbirth and stigma: the spoiling and repair of multiple social identities. Omega (Westport), 2014; 70 (2): 143-68. | Outcomes not relevant |
| Brin DJ, Chrisler JC. The Use of Rituals in Grieving for a Miscarriage or Stillbirth. Haworth Press, Brin, Deborah J., 602 11th Ave., Grinnell, IA, US, 50112 New York, NY, 2004. | Study design not relevant |
| Brown HJ. Tanner's story: a phenomenologic stance towards newborn death. Can J Nurs Res, 1997; 29 (4): 21-31. | Study design not relevant |
| Brown KK, Lewis RK, Baumgartner E, Schunn C, Maryman J, LoCurto  J. Exploring the Experience of Life Stress Among Black Women with a History of Fetal or Infant Death: a Phenomenological Study. Journal of Racial & Ethnic Health Disparities, 2017; 4 (3): 484-96. | Outcomes not relevant |
| Brunt B. Caring for bereaved parents following a stillbirth: a student midwife's perspective. MIDIRS Midwifery Digest, 2020; 30 (2): 241-43. | Study design not relevant |
| Bryan EM. The death of a newborn twin: How can support for parents be improved? Acta Geneticae Medicae et Gemellologiae: Twin Research, 1986; 35 (1): 115-18. | Outcomes not relevant |

| Reference | Reason for exclusion |
| --- | --- |
| Bucchio J, Jones N, Flores-Carter K. Support Groups for Parents Experiencing Perinatal Loss. International Journal of Childbirth Education, 2018; 33 (3): 14-17. | Outcomes not relevant |
| Budd J, Stacey T, Martin B, Roberts D, Heazell AEP. Women's experiences of being invited to participate in a case-control study of stillbirth - findings from the Midlands and North of England Stillbirth Study. BMC Pregnancy & Childbirth, 2018; 18 (1): N.PAG-N.PAG. | Perspective not relevant |
| Burden C, Bakhbakhi D, Heazell AE, Lynch M, Timlin L, Bevan C, et al. Parents' Active Role and ENgagement in The review of their Stillbirth/perinatal death 2 (PARENTS 2) study: a mixed-methods study of implementation. BMJ Open, 2021; 11 (3): e044563. | Outcomes not relevant |
| Burden C, Bakhbakhi D, Lynch M, Timlin L, Siassakos D. The PARENTS 2 Study: Parental engagement in the perinatal mortality review process-a multicentre mixed-methods study. BJOG: An International Journal of Obstetrics and Gynaecology, 2019; 126119-20. | Study design not relevant |
| Cacciatore J, Blood C, Kurker S. From “Silent Birth” to Voices Heard: Volunteering, Meaning, and Posttraumatic Growth After Stillbirth.  Illness, Crisis & Loss, 2018; 26 (1): 23-39. | Outcomes not relevant |
| Cacciatore J, Bushfield S. Stillbirth: the mother's experience and implications for improving care. J Soc Work End Life Palliat Care, 2007; 3 (3): 59-79. | Outcomes not relevant |
| Cacciatore J, DeFrain J, Jones KLC, Jones H. Stillbirth and the couple: a gender-based exploration. Journal of Family Social Work, 2008; 11 (4): 351-70. | Outcomes not relevant |
| Cacciatore J. ‘She used his name’: Provider trait mindfulness in perinatal death counselling. Estudios de Psicología, 2017; 38 (3): 639-66. | Outcomes not relevant |
| Cacciatore J. Effects of support groups on post traumatic stress responses in women experiencing stillbirth. Omega (Westport), 2007; 55  (1): 71-90. | Outcomes not relevant |
| Cacciatore J. The unique experiences of women and their families after the death of a baby. Social Work in Health Care, 2010; 49 (2): 134-48. | Outcomes not relevant |
| Callister LC. Perinatal loss: a family perspective. J Perinat Neonatal Nurs, 2006; 20 (3): 227-34; quiz 35-6. | Study design not relevant |

| Reference | Reason for exclusion |
| --- | --- |
| Camacho-Ávila M, Fernández-Sola C, Jiménez-López FR, Granero- Molina J, Fernández-Medina IM, Martínez-Artero L, et al. Experience of parents who have suffered a perinatal death in two Spanish hospitals: a qualitative study. BMC Pregnancy & Childbirth, 2019; 19 (1): 1-11. | Outcomes not relevant |
| Campbell BB. Shattered futures, mended lives: The ritualized mourning of mothers of stillborn babies. Vol. 61, ProQuest Information & Learning, 2001, pp. 3825-25. | Outcomes not relevant |
| Capitulo KL. Perinatal grief online. MCN Am J Matern Child Nurs, 2004; 29 (5): 305-11. | Outcomes not relevant |
| Capitulo KML. Ethnography of perinatal grief online. Columbia University, 2002, pp. 185 p-85 p. | Outcomes not relevant |
| Carlson R, Lammert C, O'Leary JM. THE EVOLUTION OF GROUP AND ONLINE SUPPORT FOR FAMILIES WHO HAVE EXPERIENCED PERINATAL OR NEONATAL LOSS. Illness, Crisis & Loss, 2012; 20 (3): 275-93. | Outcomes not relevant |
| Casalta Miranda AM, Otília Brites Zangão M. Mothers' experiences of fetal death. Revista de Enfermagem Referência, 2020; (3): 1-8. | Population not relevant |
| Chebsey C, Jackson S, Gleeson K, Winter C, Storey C, Hillman J, et al. Joint perspective, joint decision making; improving maternity bereavement care for stillbirth. A mixed methods multicentre study in the UK providing an in-depth understanding of maternity bereavement care. Archives of Disease in Childhood: Fetal and Neonatal Edition, 2014; 99A22. | Study design not relevant |
| Chebsey C, Jackson S, Gleeson K, Winter C, Storey C, Lewis J, et al. Joint perspective, joint decision making: Improving maternity bereavement care for stillbirth. A mixed methods multicentre study in the United Kingdom. BJOG: An International Journal of Obstetrics and Gynaecology, 2015; 122358. | Study design not relevant |
| Chebsey C, Jackson S, Lee V, Douglas T, Nutt C, White I, et al. Stillbirth-is culturally sensitive care required in the developed world? International Journal of Gynecology and Obstetrics, 2015; 131E526. | Study design not relevant |
| Chebsey C, Siassakos D, Draycott T, Winter C, Jackson S, Gleeson K, et al. Joint perspective, joint decision making; improving maternity bereavement care for stillbirth. A mixed methods multi-centre study in the UK. BJOG: An International Journal of Obstetrics and Gynaecology, 2014; 12195. | Study design not relevant |

| Reference | Reason for exclusion |
| --- | --- |
| Chebsey C, Siassakos D, Jackson S, Douglas T, Lee V, White I, et al. Multicultural views on stillbirth and maternity bereavement care. BJOG: An International Journal of Obstetrics and Gynaecology, 2014; 121116. | Study design not relevant |
| Chebsey C, Siassakos D, Jackson S, Gleeson K, Winter C, Storey C, et al. Joint perspective, joint decision making; improving maternity bereavement care for stillbirth. A mixed methods multicentre study in the UK. International Journal of Gynecology and Obstetrics, 2015; 131E253. | Study design not relevant |
| Chizhova MA. Psychological help after perinatal death as a prophylactic of reproductive problems: Contemporary situation in Russia.  Reproductive BioMedicine Online, 2010; 20S54. | Study design not relevant |
| Cholette M, Gephart SM. A Model for the Dynamics of Bereavement Caregiving. International Journal of Childbirth Education, 2012; 27 (2):  14-18. | Study design not relevant |
| Cholette ME. Exploring the meaning of the paternal experience of perinatal loss: A phenomenological study. University of Arizona, 2012,  pp. 109 p-09 p. | Population not relevant |
| Clarke J. OA56 Perinatal grief as a deeply social experience: perspectives of bereaved parents. BMJ supportive & palliative care, 2015; 5A18. | Study design not relevant |
| Colon EJ. Paternal experiences after a perinatal loss. University of South Carolina, 2008, pp. 146 p-46 p. | Study design not relevant |
| Colsen TL. Fathers and perinatal loss: Their conscious and unconscious experiences. Vol. 62, ProQuest Information & Learning, 2001, pp.  2051-51. | Outcomes not relevant |
| Conry J, Prinsloo C. Mothers' access to supportive hospital services after the loss of a baby through stillbirth or neonatal death. Health SA Gesondheid, 2008; 13 (2): 14-24. | Perspective not relevant |
| Conway P, Valentine D. Reproductive losses and grieving. Journal of Social Work & Human Sexuality, 1987; 6 (1): 43-64. | Outcomes not relevant |
| Corbet-Owen C. Women's perceptions of partner support in the context of pregnancy loss(es). South African Journal of Psychology, 2003; 33 (1): 19-27. | Setting not relevant |
| Côté-Arsenault D, Denney-Koelsch E. “Have no regrets:” Parents' experiences and developmental tasks in pregnancy with a lethal fetal diagnosis. Social Science & Medicine, 2016; 154100-09. | Population not relevant |

| Reference | Reason for exclusion |
| --- | --- |
| Côté-Arsenault D, Denney-Koelsch E. “Love Is a Choice”: Couple Responses to Continuing Pregnancy With a Lethal Fetal Diagnosis. Illness, Crisis & Loss, 2018; 26 (1): 5-22. | Population not relevant |
| Côté-Arsenault D, Denney-Koelsch E. 'My Baby Is a Person': Parents' Experiences with Life-Threatening Fetal Diagnosis. Journal of Palliative Medicine, 2011; 14 (12): 1302-08. | Population not relevant |
| Côté-Arsenault D. Weaving babies lost in pregnancy into the fabric of the family. Journal of Family Nursing, 2003; 9 (1): 23-37. | Outcomes not relevant |
| Covington SN, Theut SK. Reactions to perinatal loss: a qualitative analysis of the National Maternal and Infant Health Survey. Am J Orthopsychiatry, 1993; 63 (2): 215-22. | Study design not relevant |
| Cowan L, Wainwright CL. The death of a baby in our care: the impact on the midwife. MIDIRS Midwifery Digest, 2001; 11 (3): 313-16. | Study design not relevant |
| Crawford A, Hopkin A, Rindler M, Johnson E, Clark L, Rothwell E. Women's Experiences With Palliative Care During Pregnancy. J Obstet Gynecol Neonatal Nurs, 2021; 50 (4): 402-11. | Population not relevant |
| Crawley R, Brierley-Jones L, Gordon I, Jones E, Hinshaw K. Providing care for families who have experienced stillbirth: A qualitative, exploratory study of the views of healthcare professionals and support staff. Journal of Reproductive and Infant Psychology, 2016; 34 (4): e34. | Study design not relevant |
| Crowther ME. Communication following a stillbirth or neonatal death: room for improvement. Br J Obstet Gynaecol, 1995; 102 (12): 952-6. | Study design not relevant |
| Curtis P. Midwives' attendances at stillbirths: an oral history account. MIDIRS Midwifery Digest, 2000; 10 (4): 526-30. | Setting not relevant |
| Davidson D, Letherby G. Griefwork online: perinatal loss, lifecourse disruption and online support. Hum Fertil (Camb), 2014; 17 (3): 214-7. | Outcomes not relevant |
| Davidson D. A technology of care: Caregiver response to perinatal loss. Women's Studies International Forum, 2008; 31 (4): 278-84. | Population not relevant |
| Davies B, Limbo R, Webb NB. The grief of siblings. The Guilford Press, New York, NY, 2010. | Study design not relevant |
| Davis DL, Stewart M, Harmon RJ. Perinatal loss: providing emotional support for bereaved parents. Birth, 1988; 15 (4): 242-6. | Study design not relevant |

| Reference | Reason for exclusion |
| --- | --- |
| Dawn CM. The surviving twin: Exploring the psychological, emotional, and spiritual impacts of having experienced a death before or at birth. Vol. 64, ProQuest Information & Learning, 2004, pp. 5264-64. | Setting not relevant |
| de Andrade Alvarenga W, deMontigny F, Zeghiche S, Verdon C, Castanheira Nascimento L. Experience of hope: An exploratory research with bereaved mothers following perinatal death. Women Birth, 2021; 34 (4): e426-e34. | Population not relevant |
| de Montigny F, Beaudet L, Dumas L. A baby has died: the impact of perinatal loss on family social networks. JOGNN: Journal of Obstetric, Gynecologic & Neonatal Nursing, 1999; 28 (2): 151-56. | Population not relevant |
| DeFrain J, Martens L, Stork J, Stork W. The psychological effects of a stillbirth on surviving family members. Omega: Journal of Death and Dying, 1990; 22 (2): 81-108. | Outcomes not relevant |
| Denney-Koelsch EM, Côté-Arsenault D, Hall WJ. Feeling cared for versus experiencing added burden: Parents' interactions with health-care providers in pregnancy with a lethal fetal diagnosis. Illness, Crisis, & Loss, 2018; 26 (4): 293-315. | Population not relevant |
| Diamond RM, Roose RE. Development and Evaluation of a Peer Support Program for Parents Facing Perinatal Loss. Nurs Womens Health, 2016; 20 (2): 146-56. | Population not relevant |
| Dickerson RE. Impact of perinatal loss among adolescent parents: A phenomenological study. Vol. 72, ProQuest Information & Learning, 2012, pp. 4316-16. | Population not relevant |
| Dilts M, Lund DA. The eloquence of pain: Poetry of bereaved fathers following a perinatal loss. Baywood Publishing Co, Amityville, NY, 2001. | Study design not relevant |
| Domogalla JS, McCord J, Morse R. Rural Perinatal Loss: A Needs Assessment. Omega (Westport), 2022; 84 (4): 1045-60. | Population not relevant |
| Drake TR. 'The god of all comfort:' Experiences from a biblically-based perinatal loss support group. Vol. 71, ProQuest Information & Learning, 2011, pp. 3305-05. | Outcomes not relevant |
| Druguet M, Nuño L, Rodó C, Arévalo S, Carreras E, Gómez-Benito J. Emotional Effect of the Loss of One or Both Fetuses in a Monochorionic Twin Pregnancy. JOGNN: Journal of Obstetric, Gynecologic & Neonatal Nursing, 2018; 47 (2): 137-45. | Outcomes not relevant |

| Reference | Reason for exclusion |
| --- | --- |
| Druguet M, Nuno L, Rodo C, Arevalo S, Carreras Moratonas E, Gomez-Benito J. Influence of farewell rituals and psychological vulnerability on grief following perinatal loss in monochorionic twin pregnancy. J Matern Fetal Neonatal Med, 2019; 32 (6): 1033-35. | Outcomes not relevant |
| Due C, Obst K, Riggs DW, Collins C. Australian heterosexual women's experiences of healthcare provision following a pregnancy loss. Women Birth, 2018; 31 (4): 331-38. | Outcomes not relevant |
| Einaudi MA, Le Coz P, Malzac P, Michel F, D'Ercole C, Gire C. Parental experience following perinatal death: exploring the issues to make progress. European Journal of Obstetrics, Gynecology, & Reproductive Biology, 2010; 151 (2): 143-8. | Population not relevant |
| Ekelin M, Crang-Svalenius E, Nordstrom B, Dykes AK. Parents' experiences, reactions and needs regarding a nonviable fetus diagnosed at a second trimester routine ultrasound. J Obstet Gynecol Neonatal Nurs, 2008; 37 (4): 446-54. | Population not relevant |
| Erlandsson K, Avelin P, Saflund K, Wredling R, Radestad I. Siblings' farewell to a stillborn sister or brother and parents' support to their older children: a questionnaire study from the parents' perspective. J Child Health Care, 2010; 14 (2): 151-60. | Outcomes not relevant |
| Erlandsson K, Lindgren H, Davidsson-Bremborg A, Rådestad I. Women's premonitions prior to the death of their baby in utero and how they deal with the feeling that their baby may be unwell. Acta Obstetricia et Gynecologica Scandinavica, 2012; 91 (1): 28-33. | Setting not relevant |
| Erlandsson K, Lindgren H, Malm MC, Davidsson-Bremborg A, Radestad I. Mothers' experiences of the time after the diagnosis of an intrauterine death until the induction of the delivery: a qualitative Internet-based study. J Obstet Gynaecol Res, 2011; 37 (11): 1677-84. | Setting not relevant |
| Erlandsson K, Saflund K, Wredling R, Radestad I. Support after stillbirth and its effect on parental grief over time. J Soc Work End Life Palliat Care, 2011; 7 (2-3): 139-52. | Study design not relevant |
| Estok P, Lehman A. Perinatal death: grief support for families. Birth, 1983; 10 (1): 17-25. | Population not relevant |
| Farrales L, Douglas J, Ascher J, Nanson J, Farrales M, McComb A, et al. Bereaved parents break the silence of stillbirth Community-based participatory research project. Canadian Family Physician, 2015; 61 (2): S50. | Study design not relevant |

| Reference | Reason for exclusion |
| --- | --- |
| Fenstermacher K. Bereavement Support for Black Adolescents After Perinatal Loss...28th Annual Scientific Session, June 2-6, 2017, Baltimore, Maryland. Nursing Research, 2016; 65 (2): E60-E60. | Study design not relevant |
| Fenstermacher KH, Hupcey JE. Support for Young Black Urban Women After Perinatal Loss. MCN Am J Matern Child Nurs, 2019; 44  (1): 13-19. | Study design not relevant |
| Fenstermacher KH. Enduring to gain new perspective: a grounded theory study of the experience of perinatal bereavement in Black adolescents. Res Nurs Health, 2014; 37 (2): 135-43. | Population not relevant |
| Fenstermacher KH. Perinatal loss and bereavement in non-Hispanic Black adolescents. Pennsylvania State University, 2011, pp. 235 p-35 p. | Population not relevant |
| Fernández-Basanta S, Coronado C, Bondas T, Movilla-Fernández M-J. Primary healthcare midwives' experiences of caring for parents who have suffered an involuntary pregnancy loss: a phenomenological hermeneutic study. Midwifery, 2021; 92N.PAG-N.PAG. | Population not relevant |
| Fernandez-Basanta S, Van P, Coronado C, Torres M, Movilla- Fernandez MJ. Coping After Involuntary Pregnancy Loss: Perspectives of Spanish European Women. Omega (Westport), 2021; 83 (2): 310-24. | Population not relevant |
| Fernandez-Sola C, Camacho-Avila M, Hernandez-Padilla JM, Fernandez-Medina IM, Jimenez-Lopez FR, Hernandez-Sanchez E, et al. Impact of Perinatal Death on the Social and Family Context of the Parents. Int J Environ Res Public Health, 2020; 17 (10): 14. | Outcomes not relevant |
| Fleming V, Robb Y, Matteo C, Meier-Magistretti C. When New Life Meets Death: Three Hermeneutic Case Studies From Switzerland.  Omega (Westport), 2022; 85 (1): 204-24. | Population not relevant |
| Fogarty S. A role for massage after antenatal or neonatal loss: evaluations from a community program. Advances in Integrative Medicine, 2021; 8 (2): 129-35. | Outcomes not relevant |
| Forrest GC, Standish E, Baum JD. Support after perinatal death: a study of support and counselling after perinatal bereavement. Br Med J (Clin Res Ed), 1982; 285 (6353): 1475-9. | Population not relevant |
| Francois S. Supporting African American Mothers following a Stillbirth: Relationship Quality Matters. International Journal of Childbirth Education, 2018; 33 (4): 6-9. | Outcomes not relevant |

| Reference | Reason for exclusion |
| --- | --- |
| Furton KM. Becoming a parent without a child: The experience of losing a first pregnancy A heuristic inquiry. Vol. 78, ProQuest Information & Learning, 2017. | Population not relevant |
| Gandino G, Bernaudo A, Di Fini G, Vanni I, Veglia F. Meanings of perinatal loss: A thematic analysis of health workers' experiences.  Minerva Psichiatrica, 2016; 57 (3): 104-12. | Outcomes not relevant |
| Gandino G, Di Fini G, Bernaudo A, Paltrinieri M, Castiglioni M, Veglia  F. The impact of perinatal loss in maternity units: A psycholinguistic analysis of health professionals' reactions. J Health Psychol, 2020; 25  (5): 640-51. | Study design not relevant |
| Garcia R, Ali N, Griffiths M, Randhawa G. A qualitative study exploring the experiences of bereavement after stillbirth in pakistani, bangladeshi and white british mothers living in luton, UK. Midwifery, 2020; 91102833. | Population not relevant |
| Gardner JM. Perinatal death: uncovering the needs of midwives and nurses and exploring helpful interventions in the United States, England, and Japan. J Transcult Nurs, 1999; 10 (2): 120-30. | Study design not relevant |
| Gavrizi S, Pike J, Mak W. Understanding the Needs of Individuals Who Have Experienced Pregnancy Loss: A Retrospective Community-Based Survey. Journal of Women's Health, 2021; 101. | Study design not relevant |
| Gilbert KR, Smart LS. Coping with infant or fetal loss: The couple's healing process. Brunner/Mazel, Philadelphia, PA, 1992. | Study design not relevant |
| Glasgow C. Midwives' reflections and coping strategies around neonatal death. MIDIRS Midwifery Digest, 2017; 27 (1): 115-18. | Study design not relevant |
| Glatfelter KS. Understanding and addressing the needs of women experiencing perinatal loss leading to hospital protocol change.  Understanding & Addressing the Needs of Women Experiencing Perinatal Loss Leading to Hospital Protocol Change, 2017;1-1. | Population not relevant |
| Golan A, Leichtentritt RD. Meaning Reconstruction among Women following Stillbirth: A Loss Fraught with Ambiguity and Doubt. Health & Social Work, 2016; 41 (3): 147-54. | Outcomes not relevant |
| Gold KJ, Boggs ME, Mugisha E, Palladino CL. Internet message boards for pregnancy loss: who's on-line and why? Womens Health Issues, 2012; 22 (1): e67-72. | Study design not relevant |

| Reference | Reason for exclusion |
| --- | --- |
| Goldberg J, Rose K, Matthews O, Boles J. Little time, lasting impact: Bereaved caregiver perceptions of legacy in perinatal and infant loss. J Neonatal Perinatal Med, 2022; 15 (3): 617-26. | Population not relevant |
| Grady KL. Parenting a lone twin: When one twin dies. Boston College, 2012, pp. 149 p-49 p. | Outcomes not relevant |
| Graham MA, Thompson SC, Estrada M, Yonekura ML. Factors affecting psychological adjustment to a fetal death. Am J Obstet Gynecol, 1987; 157 (2): 254-7. | Outcomes not relevant |
| Griffin CWM. It's a birth not a procedure: An ethnographic study of intrauterine fetal death in a labor and delivery unit of an American hospital setting. Wayne State University, 2012, pp. 204 p-04 p. | Outcomes not relevant |
| Griffiths C, McAra-Couper J, Nayar S. Staying Involved "Because the Need Seems So Huge": Midwives Working With Women Living in Areas of High Deprivation. International Journal of Childbirth, 2013; 3  (4): 218-31. | Setting not relevant |
| Grubb-Phillips CA. Intrauterine fetal death: the maternal bereavement experience. J Perinat Neonatal Nurs, 1988; 2 (2): 34-44. | Study design not relevant |
| Hamama-Raz Y, Hartman H, Buchbinder E. Coping With Stillbirth Among Ultraorthodox Jewish Women. Qual Health Res, 2014; 24 (7): 923-32. | Outcomes not relevant |
| Harris Trimiar D. Understanding grief in stillbirth: A phenomenological study of African American women. Vol. 82, ProQuest Information & Learning, 2021. | Outcomes not relevant |
| Haward MF, Lorenz JM, Janvier A, Fischhoff B. Bereaved Parents: Insights for the Antenatal Consultation. Am J Perinatol, 2021. | Population not relevant |
| Hazen MA. Societal and workplace responses to perinatal loss: disenfranchised grief or healing connection. Human Relations, 2003; 56  (2): 147-66. | Outcomes not relevant |
| Heazell AE, McLaughlin MJ, Schmidt EB, Cox P, Flenady V, Khong TY, et al. A difficult conversation? The views and experiences of parents and professionals on the consent process for perinatal postmortem after stillbirth. BJOG, 2012; 119 (8): 987-97. | Study design not relevant |
| Hebert MP. Perinatal bereavement in its cultural context. Death Stud, 1998; 22 (1): 61-78. | Study design not relevant |

| Reference | Reason for exclusion |
| --- | --- |
| Henderson J, Redshaw M. Parents' experience of perinatal post-mortem following stillbirth: A mixed methods study. PLoS One, 2017; 12 (6): e0178475. | Outcomes not relevant |
| Hicks S. Early pregnancy loss in African American women: An exploratory phenomenological study. Vol. 82, ProQuest Information & Learning, 2021. | Population not relevant |
| Hill JE, White S, Hopkins Hutti M, Polivka B, Clark PR, Cooke C, et al. The Meaning, Experiences, and Behaviors of Nurses Caring for Women With a Perinatal Loss. JOGNN: Journal of Obstetric, Gynecologic & Neonatal Nursing, 2014; 43S76-S77. | Study design not relevant |
| Hillowe-Donahue CA. Maternal bereavement following late stillbirth: An exploratory study of the influence of socio-demographic factors and health care practices on the grief patterns of women experiencing 3rd trimester pregnancy loss. Vol. 68, ProQuest Information & Learning, 2007, pp. 751-51. | Study design not relevant |
| Höglund B, Rådestad I, Hildingsson I. Few women receive a specific explanation of a stillbirth - an online survey of women's perceptions and thoughts about the cause of their baby's death. BMC Pregnancy & Childbirth, 2019; 19 (1): 139-39. | Outcomes not relevant |
| Homer CSE, Malata A, Ten Hoope-Bender P. Supporting women, families, and care providers after stillbirths. Lancet, 2016; 387 (10018): 516-17. | Study design not relevant |
| Huberty J, Sullivan M, Green J, Kurka J, Leiferman J, Gold K, et al. Online yoga to reduce post traumatic stress in women who have experienced stillbirth: a randomized control feasibility trial. BMC Complementary Medicine & Therapies, 2020; 20 (1): 1-19. | Outcomes not relevant |
| Huberty JL, Coleman J, Rolfsmeyer K, Wu S. A qualitative study exploring women's beliefs about physical activity after stillbirth. BMC Pregnancy & Childbirth, 2014; 1426. | Outcomes not relevant |
| Human M, Goldstein RD, Groenewald CA, Kinney HC, Odendaal HJ, Network P. Bereaved mothers' attitudes regarding autopsy of their stillborn baby. The South African journal of obstetrics and gynaecology  : SAJOG : the journal of the South African Society of Obstetricians and Gynaecologists, 2017; 23 (3): 93-96. | Study design not relevant |
| Hunfeld JAM, Wladimiroff JW, Verhage F, Passchier J. Previous stress and acute psychological defence as predictors of perinatal grief -- an exploratory study. Social Science & Medicine, 1995; 40 (6): 829-35. | Population not relevant |

| Reference | Reason for exclusion |
| --- | --- |
| Hutti MH. A Comparison of the Caring Processes Used By Obstetric, Surgical, and Emergency Nurses When Caring for the Woman with a Fetal Loss...Proceedings of the 2015 AWHONN Convention. JOGNN: Journal of Obstetric, Gynecologic & Neonatal Nursing, 2015; 44S69- S69. | Study design not relevant |
| Ibiebele I, Flenady V, Horey D, Wilson P, Coory M, Boyle F. Autopsy consent process: What parents are saying following a stillbirth. Journal of Paediatrics and Child Health, 2015; 5128. | Study design not relevant |
| Jakubowski B, Oakley L, Duclos D. A review of the stillbirth registration process in the UK: Support and care offered to parents. BMC Pregnancy and Childbirth, 2017; 17. | Study design not relevant |
| Jones K, Smythe L. The impact on midwives of their first stillbirth. New Zealand College of Midwives Journal, 2015; (51): 17-22. | Outcomes not relevant |
| Jones K. Renegotiating father's identity following stillbirth: What and who am I? BMC Pregnancy and Childbirth, 2017; 17. | Study design not relevant |
| Jones-Peeples AE. Understanding paternal experience of perinatal loss and maternal expectations. Vol. 74, ProQuest Information & Learning, 2014. | Outcomes not relevant |
| Jordan A, Smith P, Rodham K. Bittersweet: a qualitative exploration of mothers' experiences of raising a single surviving twin. Psychol Health Med, 2018; 23 (8): 891-98. | Outcomes not relevant |
| Jørgensen ML, Prinds C, Mørk S, Hvidtjørn D. Stillbirth – transitions and rituals when birth brings death: Data from a danish national cohort seen through an anthropological lens. Scandinavian Journal of Caring Sciences, 2021. | Study design not relevant |
| Kaunonen M, Tarkka MT, Hautamaki K, Paunonen M. The staff's experience of the death of a child and of supporting the family. Int Nurs Rev, 2000; 47 (1): 46-52. | Population not relevant |
| Kavanaugh K, Hershberger P. Perinatal loss in low-income African American parents. J Obstet Gynecol Neonatal Nurs, 2005; 34 (5): 595-  605. | Population not relevant |
| Kavanaugh K, Moro TT, Savage TA, Reyes M, Wydra M. Supporting parents' decision making surrounding the anticipated birth of an extremely premature infant. J Perinat Neonatal Nurs, 2009; 23 (2): 159-  70. | Population not relevant |

| Reference | Reason for exclusion |
| --- | --- |
| Kavanaugh K, Robertson PA. Recurrent perinatal loss: a case study. Omega (Westport), 1999; 39 (2): 133-47. | Population not relevant |
| Kavanaugh K, Trier D, Korzec M. Social support following perinatal loss. J Fam Nurs, 2004; 10 (1): 70-92. | Outcomes not relevant |
| Kempson D, Murdock V. Memory keepers: a narrative study on siblings never known. Death Stud, 2010; 34 (8): 738-56. | Setting not relevant |
| Kennedy J, Matthews A, Abbott L, Dert J, Weaver G, Shenker N. Lactation following bereavement: how can midwives support women to make informed choices? MIDIRS Midwifery Digest, 2017; 27 (4): 497-  501. | Study design not relevant |
| Kesbiç Ş, Boz İ. Experiences of perinatal nurses regarding compassion fatigue and compassion satisfaction: A phenomenological study. Journal of Psychosomatic Obstetrics & Gynecology, 2021. | Outcomes not relevant |
| Kimble DL. Neonatal death: a descriptive study of fathers' experiences. Neonatal Netw, 1991; 9 (8): 45-9. | Population not relevant |
| Kirshner S. A hermeneutical phenomenological study of Jewish orthodox women who experienced miscarriage, stillbirth, and neonatal death. Vol. 79, ProQuest Information & Learning, 2018. | Outcomes not relevant |
| Kitson C. Fathers experienced stillbirth as a waste of life and needed to protect their partners and express grief in their own way. Evidence Based Nursing, 2002;61-61. | Study design not relevant |
| Kothari A, Bruxner G, Callaway L, Dulhunty JM. "It's a lot of pain you've got to hide": a qualitative study of the journey of fathers facing traumatic pregnancy and childbirth. BMC Pregnancy Childbirth, 2022; 22 (1): 434. | Population not relevant |
| Kristvik E. The precarious space for mourning: Sick leave as an ambiguous topic in bereaved parents’ accounts of the return to everyday life after reproductive loss. Culture, Medicine, and Psychiatry: An International Journal of Cross-Cultural Health Research, 2021. | Study design not relevant |
| Kurz MR. When Death Precedes Birth: The Embodied Experiences of Women with a History of Miscarriage or Stillbirth—A Phenomenological Study Using Artistic Inquiry. American Journal of Dance Therapy, 2020; 42 (2): 194-222. | Population not relevant |

| Reference | Reason for exclusion |
| --- | --- |
| Laing RE, Fetherston CM, Morrison P. Responding to catastrophe: A case study of learning from perinatal death in midwifery practice.  Women Birth, 2020; 33 (6): 556-65. | Population not relevant |
| Lang A, Fleiszer AR, Duhamel F, Sword W, Gilbert KR, Corsini-Munt  S. Perinatal loss and parental grief: the challenge of ambiguity and disenfranchised grief. Omega (Westport), 2011; 63 (2): 183-96. | Outcomes not relevant |
| Lang A, Gottlieb LN, Amsel R. Predictors of husbands' and wives' grief reactions following infant death: the role of marital intimacy. Death Stud, 1996; 20 (1): 33-57. | Outcomes not relevant |
| LaRoche C, Lalinec-Michaud M, Engelsmann F, Fuller N, Copp M, McQuade-Soldatos L, et al. Grief reactions to perinatal death--a follow- up study. Can J Psychiatry, 1984; 29 (1): 14-9. | Outcomes not relevant |
| Lathrop A, Vandevusse L. Affirming motherhood: validation and invalidation in women's perinatal hospice narratives. Birth, 2011; 38 (3): 256-65. | Setting not relevant |
| Lathrop A. A narrative analysis of perinatal hospice stories. 2010;216 p- 16 p. | Setting not relevant |
| Lauterbach SS. In another world: a phenomenological perspective and discovery of meaning in mothers' experience of death of a wished-for baby. COLUMBIA UNIVERSITY TEACHERS COLLEGE, 1992, pp. 172 p-72 p. | Setting not relevant |
| Lauterbach SS. In another world: a phenomenological perspective and discovery of meaning in mothers' experience with death of a wished-for baby. Pennsylvania Nurse, 1993; 48 (11): 18-18. | Setting not relevant |
| Lauterbach SS. In another world: five years later -- a phenomenological nursing inquiry into meanings, 'essences' of mothers' lived experience with perinatal death of a wished-for baby unfolding over time.  International Journal for Human Caring, 2002; 6 (1): 17-24. | Setting not relevant |
| Lauterbach SS. Phenomenological silence surrounding infant death. International Journal for Human Caring, 2003; 7 (2): 38-43. | Study design not relevant |
| Lee C. “There was never going to be a happy ending”: Experiencing late pregnancy loss in Australia. Journal of Reproductive and Infant Psychology, 2011; 29 (3): e10. | Outcomes not relevant |
| Leichtentritt RD, Mahat-Shamir M. Mothers' Continuing Bond With the Baby: The Case of Feticide. Qual Health Res, 2017; 27 (5): 665-76. | Population not relevant |

| Reference | Reason for exclusion |
| --- | --- |
| Lemmer CM. Mothers' and fathers' experiences of perinatal bereavement. UNIVERSITY OF UTAH, 1988, pp. 287 p-87 p. | Outcomes not relevant |
| Lemmer CM. Parental perceptions of caring following perinatal bereavement... including commentary by Boyd P and Forrest D with author response. Western Journal of Nursing Research, 1991; 13 (4): 475-93. | Population not relevant |
| Lemos LFS, da Cunha ACB. Death in the maternity hospital: How health professionals deal with the loss. Psicologia em Estudo, 2015; 20  (1): 13-22. | Setting not relevant |
| Liisa AA, Marja-Terttu T, Paivi AK, Marja K. Health care personnel's experiences of a bereavement follow-up intervention for grieving parents. Scand J Caring Sci, 2011; 25 (2): 373-82. | Outcomes not relevant |
| Lindberg CE. The grief response to mid-trimester fetal loss. J Perinatol, 1992; 12 (2): 158-63. | Study design not relevant |
| Linde A, Pettersson K, Rådestad I. Women's Experiences of Fetal Movements before the Confirmation of Fetal Death--Contractions Misinterpreted as Fetal Movement. Birth: Issues in Perinatal Care, 2015; 42 (2): 189-94. | Perspective not relevant |
| Littlemore J, McGuinness S, Fuller D, Kuberska K, Turner S. Death before birth: understanding, informing and supporting the choices made by people who have experienced miscarriage, termination, and stillbirth. MIDIRS Midwifery Digest, 2020; 30 (2): 251-55. | Study design not relevant |
| Lizcano Pabon LDM, Moreno Fergusson ME, Palacios AM. Experience of Perinatal Death From the Father's Perspective. Nurs Res, 2019; 68  (5): E1-E9. | Setting not relevant |
| Lockton J, Due C, Oxlad M. Love, Listen and Learn: Grandmothers' Experiences of Grief Following Their Child's Pregnancy Loss. Women & Birth, 2020; 33 (4): 401-07. | Population not relevant |
| Lockwood S, Lewis IC. Management of grieving after stillbirth. Med J Aust, 1980; 2 (6): 308-11. | Study design not relevant |
| Lovell A. Some questions of identity: Late miscarriage, stillbirth and perinatal loss. Social Science & Medicine, 1983; 17 (11): 755-61. | Study design not relevant |
| Lukas ML. Paternal grief, mourning, and adaptation following perinatal loss. Vol. 59, ProQuest Information & Learning, 1999, pp. 5169-69. | Outcomes not relevant |

| Reference | Reason for exclusion |
| --- | --- |
| Lundqvist A, Nilstun T, Dykes A. Both empowered and powerless: mothers' experiences of professional care when their newborn dies. Birth: Issues in Perinatal Care, 2002; 29 (3): 192-99. | Population not relevant |
| MacConnell G, Aston M, Randel P, Zwaagstra N. Nurses' experiences providing bereavement follow-up: an exploratory study using feminist poststructuralism. Journal of Clinical Nursing (John Wiley & Sons, Inc.), 2013; 22 (7): 1094-102. | Setting not relevant |
| Malacrida C. Complicating mourning: the social economy of perinatal death. Qualitative Health Research, 1999; 9 (4): 504-19. | Population not relevant |
| Malacrida CA. Perinatal death: helping parents find their way. Journal of Family Nursing, 1997; 3 (2): 130-48. | Population not relevant |
| Malm MC, Lindgren H, Radestad I. Losing contact with one's unborn baby--mothers' experiences prior to receiving news that their baby has died in utero. Omega - Journal of Death & Dying, 2010; 62 (4): 353-67. | Perspective not relevant |
| Marek MJ. Cultural differences in perinatal loss: the Latino family experience. Communicating Nursing Research, 2007; 40539-39. | Outcomes not relevant |
| Maria Pastor Montero S, Manuel Romero Sanchez J, Hueso Montoro C, Lillo Crespo M, Gema Vacas Jaén A, Belén Rodriguez Tirado M. Experiences with perinatal loss from the health professionals' perspective. Revista Latino-Americana de Enfermagem (RLAE), 2011; 19 (6): 1405-12. | Study design not relevant |
| Martin CJH, Patterson J, Paterson C, Welsh N, Dougall N, Karatzias T, et al. ICD-11 complex Post Traumatic Stress Disorder (CPTSD) in parents with perinatal bereavement: Implications for treatment and care. Midwifery, 2021; 96102947. | Outcomes not relevant |
| Martin CJH, Robb Y, Forrest E. An exploratory qualitative analysis of student midwives views of teaching methods that could build their confidence to deliver perinatal bereavement care. Nurse Education Today, 2016; 3999-103. | Outcomes not relevant |
| McCarthy MR. Gender differences in reactions to perinatal loss: A qualitative study of couples. Vol. 62, ProQuest Information & Learning, 2002, pp. 3809-09. | Outcomes not relevant |
| McCloskey E, Cohen A, Blencowe H. Factors contributing to bereaved parents of stillbirth consent to post-mortem examination and reflections of their decision: A qualitative analysis. BMC Pregnancy and Childbirth, 2017; 17. | Study design not relevant |

| Reference | Reason for exclusion |
| --- | --- |
| McCool W, Guidera M, Stenson M, Dauphinee L. The pain that binds us: midwives' experiences of loss and adverse outcomes around the world. Health Care for Women International, 2009; 30 (11): 1003-13. | Outcomes not relevant |
| McCreight BS. A grief ignored: narratives of pregnancy loss from a male perspective. Sociol Health Illn, 2004; 26 (3): 326-50. | Outcomes not relevant |
| McCreight BS. Perinatal grief and emotional labour: a study of nurses' experiences in gynae wards. Int J Nurs Stud, 2005; 42 (4): 439-48. | Population not relevant |
| McGuinness D, Coughlan B, Butler M. An exploration of the experiences of mothers as they suppress lactation following late miscarriage, stillbirth or neonatal death. Evidence Based Midwifery, 2014; 12 (2): 65-70. | Outcomes not relevant |
| Meaney S, Corcoran P, O'Donoghue K. Death of one twin during the perinatal period: A qualitative study. International Journal of Gynecology and Obstetrics, 2015; 131E116. | Outcomes not relevant |
| Meaney S, Corcoran P, O'Donoghue K. Death of One Twin during the Perinatal Period: An Interpretative Phenomenological Analysis. J Palliat Med, 2017; 20 (3): 290-93. | Outcomes not relevant |
| Mickeviciute DE, Breidokiene R, Subaciute G, Lesinskiene S. Support for families dealing with perinatal and postnatal loss and bereavement. Neuropsychiatrie de l'Enfance et de l'Adolescence, 2012; 60 (5): S233. | Study design not relevant |
| Mills TA, Ricklesford C, Heazell AE, Cooke A, Lavender T. Marvellous to mediocre: findings of national survey of UK practice and provision of care in pregnancies after stillbirth or neonatal death. BMC Pregnancy Childbirth, 2016; 16101. | Setting not relevant |
| Mills TA. Improving support in pregnancy after stillbirth or neonatal death: IMPs study. BMC Pregnancy and Childbirth, 2015; 15. | Study design not relevant |
| Morton PA. Perinatal loss and the replacement child: The emotional limits of reproductive technology. Vol. 57, ProQuest Information & Learning, 1997, pp. 4133-33. | Outcomes not relevant |
| Moulder C. Clinical. Late pregnancy loss: issues in hospital care. British Journal of Midwifery, 1999; 7 (4): 244-47. | Population not relevant |
| Murphy M, Donoghue KO, Savage E, Leahy-Warren P. Stillbirth: The diagnosis of life-limiting fetal conditions and its effect on pregnancy after loss. Journal of Pain and Symptom Management, 2016; 52 (6): e70. | Study design not relevant |

| Reference | Reason for exclusion |
| --- | --- |
| Murphy M, Savage E, Donoghue KO, Leahy-Warren P. Journey of loss: The lived experiences of couples' journeys from stillbirth to subsequent pregnancy. BMC Pregnancy and Childbirth, 2017; 17. | Study design not relevant |
| Murphy S. 'I'd failed to produce a baby and I'd failed to notice when the baby was in distress': The social construction of bereaved motherhood. Women's Studies International Forum, 2019; 7435-41. | Outcomes not relevant |
| Murphy S. Reclaiming a moral identity: stillbirth, stigma and ‘moral mothers’. Midwifery, 2012; 28 (4): 476-80. | Outcomes not relevant |
| Murphy SL. Finding the positive in loss: stillbirth and its potential for parental empowerment. Bereavement Care, 2012; 31 (3): 98-103. | Study design not relevant |
| Nallen K. Midwives' needs in relation to the provision of bereavement support to parents affected by perinatal death. Part one. MIDIRS Midwifery Digest, 2006; 16 (4): 537-42. | Study design not relevant |
| Nallen K. Neonatal and infancy. Midwives' needs in relation to the provision of bereavement support to parents affected by perinatal death. Part two. MIDIRS Midwifery Digest, 2007; 17 (1): 105-12. | Outcomes not relevant |
| Ng C, Newbold KB. Health care providers' perspectives on the provision of prenatal care to immigrants. Cult Health Sex, 2011; 13 (5): 561-74. | Population not relevant |
| Nicol MT, Tompkins JR, Campbell NA, Syme GJ. Maternal grieving response after perinatal death. Med J Aust, 1986; 144 (6): 287-9. | Study design not relevant |
| Noble-Carr D, Carroll K, Waldby C. Mapping Hospital-Based Lactation Care Provided to Bereaved Mothers: A Basis for Quality Improvement. Breastfeed Med, 2021; 16 (10): 779-89. | Outcomes not relevant |
| Nordlund E, BÃ¶rjesson A, Cacciatore J, Pappas C, Randers I, RÃ¥destad I. When a baby dies: Motherhood, psychosocial care and negative affect. British Journal of Midwifery, 2012; 20 (11): 780-84. | Outcomes not relevant |
| Nurse NJ. Managing Ambiguity: Nurses Caring for the Mother of a Stillborn Baby. Managing Ambiguity: Nurses Caring For The Mother Of A Stillborn Baby, 2018;1-1. | Outcomes not relevant |
| Nurse-Clarke N, DiCicco-Bloom B, Limbo R. Application of Caring Theory to Nursing Care of Women Experiencing Stillbirth. MCN Am J Matern Child Nurs, 2019; 44 (1): 27-32. | Outcomes not relevant |

| Reference | Reason for exclusion |
| --- | --- |
| Nuzum D, Meaney S, O'Donoghue K. The Place of Faith for Consultant Obstetricians Following Stillbirth: A Qualitative Exploratory Study. J Relig Health, 2016; 55 (5): 1519-28. | Outcomes not relevant |
| Nuzum D, Meaney S, O'Donoghue K. The provision of spiritual and pastoral care following stillbirth in Ireland: a mixed methods study. BMJ Support Palliat Care, 2016; 6 (2): 194-200. | Perspective not relevant |
| Nuzum D, Meaney S, O'Donoghue K. The Spiritual and Theological Challenges of Stillbirth for Bereaved Parents. J Relig Health, 2017; 56  (3): 1081-95. | Outcomes not relevant |
| Nuzum D, Meaney S, O'Donohue K. Communication skills in Obstetrics: what can we learn from bereaved parents? Irish Medical Journal, 2017; 110 (2): 512. | Outcomes not relevant |
| O’Leary J, Warland J, Parker L. Bereaved Parents’ Perception of the Grandparents’ Reactions to Perinatal Loss and the Pregnancy That Follows. Journal of Family Nursing, 2011; 17 (3): 330-56. | Outcomes not relevant |
| Obst KL, Due C. Australian men's experiences of support following pregnancy loss: A qualitative study. Midwifery, 2019; 701-6. | Setting not relevant |
| Obst KL, Due C. Men's grief and support following pregnancy loss: A qualitative investigation of service providers' perspectives. Death Stud, 2021; 45 (10): 772-80. | Outcomes not relevant |
| Ogunbanja Y. STILLBIRTH AND SIDS: SUPPORTING PARENTS.  Community Practitioner, 2020; 93 (4): 44-47. | Outcomes not relevant |
| Okanaga M, Okamura H. How midwives grow as professions through perinatal loss care. Journal of Paediatrics and Child Health, 2012; 4892. | Study design not relevant |
| O'Leary J, Thorwick C. Fathers' perspectives during pregnancy, postperinatal loss. J Obstet Gynecol Neonatal Nurs, 2006; 35 (1): 78-86. | Population not relevant |
| O'Leary JM. Pregnancy and infant loss: supporting parents and their children. Zero to Three, 2007; 27 (6): 42-49. | Study design not relevant |
| Oreg A. Philanthropic giving in times of personal loss: Bereaved mothers’ donation of their human milk to nonprofit milk banks. Vol. 81, ProQuest Information & Learning, 2020. | Outcomes not relevant |
| Oreg A. The grief ritual of extracting and donating human milk after perinatal loss. Soc Sci Med, 2020; 265113312. | Population not relevant |

| Reference | Reason for exclusion |
| --- | --- |
| Origlia Ikhilor P, Hasenberg G, Kurth E, Stocker Kalberer B, Cignacco E, Pehlke-Milde J. Barrier-free communication in maternity care of allophone migrants: BRIDGE study protocol. J Adv Nurs, 2018; 74 (2): 472-81. | Study design not relevant |
| Paris GF, de Montigny F, Carvalho MDdB, Pelloso SM. Coping with stillbirth from the perspective of the mother: a time-series analysis.  Online Brazilian Journal of Nursing, 2014; 13386-88. | Setting not relevant |
| Paris GF, Montigny F, Pelloso SM. Professional practice in caring for maternal grief in the face of stillbirth in two countries. Rev Bras Enferm, 2021; 74 (3): e20200253. | Setting not relevant |
| Parviainen K, Kaunonen M, Aho AL. Parents' experiences about individual peer support after the death of a child. Hoitotiede, 2012; 24  (2): 150-62. | Language not relevant |
| Pastor-Montero SM, Romero-Sanchez JM, Paramio-Cuevas JC, Hueso- Montoro C, Paloma-Castro O, Lillo-Crespo M, et al. Tackling perinatal loss, a participatory action research approach: research protocol. J Adv Nurs, 2012; 68 (11): 2578-85. | Study design not relevant |
| Pearson T, Obst K, Due C. Culturally and linguistically diverse men's experiences of support following perinatal death: A qualitative study. J Clin Nurs, 2022; 2121. | Population not relevant |
| Pector EA. How bereaved multiple-birth parents cope with hospitalization, homecoming, disposition for deceased, and attachment to survivors. Journal of Perinatology, 2004; 24 (11): 714-22. | Population not relevant |
| Pector EA. Views of bereaved multiple-birth parents on life support decisions, the dying process, and discussions surrounding death. J Perinatol, 2004; 24 (1): 4-10. | Population not relevant |
| Peel E. Pregnancy loss in lesbian and bisexual women: an online survey of experiences. Hum Reprod, 2010; 25 (3): 721-7. | Population not relevant |
| Peelen J. Reversing the past: monuments for stillborn children. Mortality, 2009; 14 (2): 173-86. | Study design not relevant |
| Pena MA. Men's experience of perinatal loss. Vol. 70, ProQuest Information & Learning, 2009, pp. 700-00. | Population not relevant |
| Pilkington FB. The lived experience of grieving the loss of an important other. Nurs Sci Q, 1993; 6 (3): 130-9. | Perspective not relevant |

| Reference | Reason for exclusion |
| --- | --- |
| Pollock D, Pearson E, Cooper M, Ziaian T, Foord C, Warland J. Voices of the unheard: A qualitative survey exploring bereaved parents experiences of stillbirth stigma. Women Birth, 2020; 33 (2): 165-74. | Outcomes not relevant |
| Pollock D, Warland J, Ziaian T, Pearson E, Cooper M. A pilot study exploring stillbirth stigma experiences in Australia and adapting and validating a stigma scale. BMC Pregnancy and Childbirth, 2017; 17. | Study design not relevant |
| Popoola T, Skinner J, Woods M. Exploring the Social Networks of Women Bereaved by Stillbirth: A Descriptive Qualitative Study. J Pers Med, 2021; 11 (11): 21. | Setting not relevant |
| Power A, Atkinson S, Noonan M. "Stranger in a mask" midwives' experiences of providing perinatal bereavement care to parents during the COVID-19 pandemic in Ireland: A qualitative descriptive study. Midwifery, 2022; 111103356. | Outcomes not relevant |
| Priday A, McAra-Couper J. A Successful Midwifery Model for a High Deprivation Community in New Zealand: A Mixed Methods Study. International Journal of Childbirth, 2016; 6 (2): 78-92. | Setting not relevant |
| Quinn C. Creating and maintaining compassionate relationships with bereaved parents after perinatal death. British Journal of Midwifery, 2016; 24 (8): 562-66. | Study design not relevant |
| Radestad I, Listermar KH. Perinatal palliative care after a stillbirth - Midwives experiences of using Cubitus baby. BMC Pregnancy and Childbirth, 2017; 17. | Study design not relevant |
| Radestad I, Saflund K, Wredling R, Onelöv E, Steineck G. Holding a stillborn baby: mothers' feelings of tenderness and grief. British Journal of Midwifery, 2009; 17 (3): 178-80. | Study design not relevant |
| Rådestad I, Westerberg A, Ekholm A, Davidsson-Bremborg A, Erlandsson K. Evaluation of care after stillbirth in Sweden based on mothers' gratitude. British Journal of Midwifery, 2011; 19 (10): 646-52. | Study design not relevant |
| Rajan L, Oakley A. No pills for the heartache: The importance of social support for women who suffer pregancy loss. Journal of Reproductive and Infant Psychology, 1993; 11 (2): 75-87. | Population not relevant |
| Rajan L. 'Not just me dreaming': parents mourning pregnancy loss. Health Visitor, 1992; 65 (10): 354-57. | Outcomes not relevant |
| Rajan L. Social isolation and support in pregnancy loss. Health Visit, 1994; 67 (3): 97-101. | Outcomes not relevant |

| Reference | Reason for exclusion |
| --- | --- |
| Ramirez FD, Bogetz JF, Kufeld M, Yee LM. Professional Bereavement Photography in the Setting of Perinatal Loss: A Qualitative Analysis.  Glob Pediatr Health, 2019; 62333794X19854941. | Outcomes not relevant |
| Rankin J, Bush J, Bell R, Cresswell P, Renwick M. Impacts of participating in confidential enquiry panels: a qualitative study. BJOG, 2006; 113 (4): 387-92. | Outcomes not relevant |
| Read S, Stewart C, Cartwright P, Meigh S. Professional issues. Psychological support for perinatal trauma and loss. British Journal of Midwifery, 2003; 11 (8): 484-88. | Study design not relevant |
| Redshaw M, Henderson J, Bevan C. 'This is time we'll never get back': a qualitative study of mothers' experiences of care associated with neonatal death. BMJ Open, 2021; 11 (9): e050832. | Population not relevant |
| Redshaw M, Henderson J. Care associated with stillbirth for the most disadvantaged women: A multi‐method study of care in England. Birth: Issues in Perinatal Care, 2018; 45 (3): 275-85. | Study design not relevant |
| Remillard KIM. Turning pain into privilege. Nursing, 2003; 33 (2): 32hn4-32hn6. | Study design not relevant |
| Reyes MR. Women with Correctable Fetal Anomaly Participating in Perinatal Team Counseling: An Exploratory Study. University of Illinois at Chicago, 2014, pp. 104 p-04 p. | Outcomes not relevant |
| Richards D. Abigail's story. Pract Midwife, 2011; 14 (3): 13-5. | Study design not relevant |
| Richards J, Graham R, Embleton ND, Campbell C, Rankin J. Mothers' perspectives on the perinatal loss of a co-twin: a qualitative study. BMC Pregnancy Childbirth, 2015; 15 (1): 143. | Setting not relevant |
| Richards J, Graham RH, Embleton ND, Rankin J. Health professionals' perspectives on bereavement following loss from a twin pregnancy: a qualitative study. J Perinatol, 2016; 36 (7): 529-32. | Population not relevant |
| Richardson R. The voices of bereaved parents: Survey feedback following perinatal death. Journal of Paediatrics and Child Health, 2011; 4734. | Study design not relevant |
| Riggs DW, Pearce R, Pfeffer CA, Hines S, White FR, Ruspini E. Men, trans/masculine, and non-binary people's experiences of pregnancy loss: an international qualitative study. BMC Pregnancy Childbirth, 2020; 20  (1): 482. | Population not relevant |

| Reference | Reason for exclusion |
| --- | --- |
| Rocio Guzman I. Care of the Bereaved Mother. Care Of The Bereaved Mother, 2018;1-1. | Outcomes not relevant |
| Roehrs C, Masterson A, Alles R, Witt C, Rutt P. Caring for families coping with perinatal loss. J Obstet Gynecol Neonatal Nurs, 2008; 37  (6): 631-9. | Outcomes not relevant |
| Romney J, Fife ST, Sanders D, Behrens S. Treatment of Couples Experiencing Pregnancy Loss: Reauthoring Loss from a Narrative Perspective. Journal of Family Psychotherapy, 2021; 32 (2): 134-52. | Outcomes not relevant |
| Rondinelli J, Long K, Seelinger C, Crawford CL, Valdez R. Factors related to nurse comfort when caring for families experiencing perinatal loss: evidence for bereavement program enhancement. J Nurses Prof Dev, 2015; 31 (3): 158-63. | Study design not relevant |
| Roose R, Mirecki RM, Blanford C. Parents Supporting Parents: Implementing a Peer Parent Program for Perinatal Loss. JOGNN: Journal of Obstetric, Gynecologic & Neonatal Nursing, 2014; 43S46- S46. | Setting not relevant |
| Roose RE, Blanford CR. Perinatal grief and support spans the generations: parents' and grandparents' evaluations of an intergenerational perinatal bereavement program. J Perinat Neonatal Nurs, 2011; 25 (1): 77-85. | Outcomes not relevant |
| Rowe J, Clyman R, Green C, Mikkelsen C, Haight J, Ataide L. Follow- up families who experience a perinatal death. Pediatrics, 1978; 62 (2): 166-70. | Population not relevant |
| Sani L, Laurenti Dimanche AC, Bacque MF. Angels in the Clouds: Stillbirth and Virtual Cemeteries on 50 YouTube Videos. Omega (Westport), 2021; 82 (4): 587-608. | Population not relevant |
| Sas C, Whittaker S, Zimmerman J. Design for rituals of letting go: An embodiment perspective on disposal practices informed by grief therapy. ACM Transactions on Computer-Human Interaction, 2016; 23  (4): 1-37. | Outcomes not relevant |
| Sauvegrain P, Zeitlin J. Investigating the benefits and challenges of including bereaved women in research: a multifaceted perinatal audit in a socially disadvantaged French district. BMJ Open, 2020; 10 (9): e034715. | Outcomes not relevant |
| Sawicka M. Searching for a narrative of loss: Interactional ordering of ambiguous grief. Symbolic Interaction, 2017; 40 (2): 229-46. | Outcomes not relevant |

| Reference | Reason for exclusion |
| --- | --- |
| Schirmann A, Boyle F, Horey D, Rowlands I, Flenady V. Clinicians' view of parents' decision-making needs for autopsy consent after stillbirth. Journal of Paediatrics and Child Health, 2019; 5599. | Study design not relevant |
| Schmid LS. The lived experience of grandparents who have lost a grandchild from perinatal death: A phenomenological study. University of Cincinnati, 2000, pp. 281 p-81 p. | Population not relevant |
| Schott J, Henley A. After a late miscarriage, stillbirth or neonatal death. J Fam Health Care, 2010; 20 (4): 116-8. | Study design not relevant |
| Schott J, Henley A. After a stillbirth -- offering choices, creating memories. British Journal of Midwifery, 2009; 17 (12): 798-801. | Study design not relevant |
| Schreiber H. Parents experiencing a perinatal death found that their interactions with social institutions devalued their loss [commentary on Malacrida C. Complicating mourning: the social economy of perinatal death. QUAL HEALTH RES 1999 Jul;9(4):504-19]. Evidence Based Nursing, 2000;27-27. | Study design not relevant |
| Scott LF, Shieh C, Umoren RA, Conard T. Care Experiences of Women Who Used Opioids and Experienced Fetal or Infant Loss. JOGNN: Journal of Obstetric, Gynecologic & Neonatal Nursing, 2017; 46 (6): 846-56. | Population not relevant |
| Serafim TC, Camilo BHN, Carizani MR, Gervasio MG, Carlos DM, Salim NR. Attention to women in situation of intrauterine fetal death: experiences of health professionals. Rev Gaucha Enferm, 2021; 42e20200249. | Setting not relevant |
| Sereshti M, Nahidi F, Simbar M, Bakhtiari M, Zayeri F. An Exploration of the Maternal Experiences of Breast Engorgement and Milk Leakage after Perinatal Loss. Glob J Health Sci, 2016; 8 (9): 53876. | Setting not relevant |
| Sheehy A, Baird K. A qualitative study of early career Australian midwives' encounters with perinatal grief, loss and trauma. Women Birth, 2022; 3131. | Outcomes not relevant |
| Siassakos D, Jackson S, Gleeson K, Chebsey C, Ellis A, Storey C, et al. All bereaved parents are entitled to good care after stillbirth: a mixed- methods multicentre study (INSIGHT). BJOG: An International Journal of Obstetrics & Gynaecology, 2018; 125 (2): 160-70. | Study design not relevant |
| Silverio SA, Easter A, Storey C, Jurkovic D, Sandall J, Collaboration PG. Preliminary findings on the experiences of care for parents who | Outcomes not relevant |

| Reference | Reason for exclusion |
| --- | --- |
| suffered perinatal bereavement during the COVID-19 pandemic. BMC Pregnancy Childbirth, 2021; 21 (1): 840. |  |
| Simpson R, Bor R. 'I'm not picking up a heart-beat': Experiences of sonographers giving bad news to women during ultrasound scans. British Journal of Medical Psychology, 2001; 74 (2): 255-72. | Population not relevant |
| Simpson RT. The experience and efficacy of critical incident stress debriefing for couples after perinatal loss. Vol. 65, ProQuest Information & Learning, 2004, pp. 1564-64. | Outcomes not relevant |
| Smart LS. The marital helping relationship following pregnancy loss and infant death. Journal of Family Issues, 1992; 13 (1): 81-98. | Population not relevant |
| Smith L. Developing a pathway for women bereaved during pregnancy. Pract Midwife, 2009; 12 (9): 14-5. | Study design not relevant |
| Smith P, Vasileiou K, Jordan A. Healthcare professionals' perceptions and experiences of using a cold cot following the loss of a baby: a qualitative study in maternity and neonatal units in the UK. BMC Pregnancy & Childbirth, 2020; 20 (1): 175-75. | Outcomes not relevant |
| Smith S. Goodbye, my sweet love. Pract Midwife, 2009; 12 (9): 23-4. | Study design not relevant |
| Soto M. Anticipatory guidance: A hospital-based intervention for adolescents with perinatal loss. Child & Adolescent Social Work Journal, 2011; 28 (1): 49-62. | Study design not relevant |
| Sparshott M. The spirituality of babies: respecting the religious beliefs of bereaved parents. Journal of Neonatal Nursing, 2004; 10 (5): 152-55. | Study design not relevant |
| St John A, Cooke M, Goopy S. Shrouds of silence: three women's stories of prenatal loss. Aust J Adv Nurs, 2006; 23 (3): 8-12. | Setting not relevant |
| Stephens L, Navin C, Thomas S, Sripada S, Church E, Tower C, et al. Improving quality of care in pregnancies after stillbirth-an improvement science project in two UK maternity hospitals. BMC Pregnancy and Childbirth, 2017; 17. | Study design not relevant |
| Stolberg J. 'Leaving footprints on our hearts' -- how can midwives provide meaningful emotional support after a perinatal death? MIDIRS Midwifery Digest, 2011; 21 (1): 7-13. | Outcomes not relevant |
| Stringham JG, Riley JH, Ross A. Silent birth: mourning a stillborn baby. Soc Work, 1982; 27 (4): 322-7. | Study design not relevant |

| Reference | Reason for exclusion |
| --- | --- |
| Sun JC, Rei W, Chang MY, Sheu SJ. Care and management of stillborn babies from the parents' perspective: A phenomenological study. J Clin Nurs, 2022; 31 (7-8): 860-68. | Setting not relevant |
| Sutan R, Miskam HM. Psychosocial impact of perinatal loss among Muslim women. BMC Women's Health, 2012; 12 (1): 15-15. | Setting not relevant |
| Swanson PB, Kane RT, Pearsall-Jones JG, Swanson CF, Croft ML. How couples cope with the death of a twin or higher order multiple. Twin Research and Human Genetics, 2009; 12 (4): 392-402. | Outcomes not relevant |
| Symes J. What comfort for this grief? Coping with perinatal bereavement. Professional Nurse, 1991; 6 (8): 437-41. | Study design not relevant |
| Tanacıoğlu-Aydın B, Erdur-Baker Ö. Pregnancy loss experiences of couples in a phenomenological study: Gender differences within the turkish sociocultural context. Death Studies, 2021. | Setting not relevant |
| Testoni I, Bregoli J, Pompele S, Maccarini A. Social Support in Perinatal Grief and Mothers' Continuing Bonds: A Qualitative Study With Italian Mourners. Affilia: Journal of Women & Social Work, 2020; 35 (4): 485-  502. | Outcomes not relevant |
| Thompson SJP. Framing history: The meaning parents ascribe to bereavement photographs received following perinatal loss. Vol. 62, ProQuest Information & Learning, 2002, pp. 3558-58. | Outcomes not relevant |
| Thornton R, Nicholson P, Harms L. Creating Evidence: Findings from a Grounded Theory of Memory-Making in Neonatal Bereavement Care in Australia. J Pediatr Nurs, 2020; 5329-35. | Population not relevant |
| Tomlinson AJ, Martindale E, Bancroft K, Heazell A. Improved management of stillbirth using a care pathway. International Journal of Health Governance, 2018; 23 (1): 18-37. | Study design not relevant |
| Toohill J, Fenwick J, Sidebotham M, Gamble J, Creedy DK. Trauma and fear in Australian midwives. Women Birth, 2019; 32 (1): 64-71. | Outcomes not relevant |
| Tovey R, Turner S. Stillbirth memento photography. J Vis Commun Med, 2020; 43 (1): 2-16. | Outcomes not relevant |
| Tseng YF, Hsu MT, Hsieh YT, Cheng HR. The meaning of rituals after a stillbirth: A qualitative study of mothers with a stillborn baby. J Clin Nurs, 2018; 27 (5-6): 1134-42. | Setting not relevant |

| Reference | Reason for exclusion |
| --- | --- |
| Ujda RM, Bendiksen R. Health care provider support and grief after perinatal loss: A qualitative study. Illness, Crisis, & Loss, 2000; 8 (3): 265-85. | Population not relevant |
| Väisänen L. Family grief and recovery process when a baby dies. Psychiatria Fennica, 1998; 29163-74. | Population not relevant |
| Van P, Meleis AI. Coping with grief after involuntary pregnancy loss: perspectives of African American women. J Obstet Gynecol Neonatal Nurs, 2003; 32 (1): 28-39. | Population not relevant |
| Van P. Breaking the silence of African American women: healing after pregnancy loss. Health Care Women Int, 2001; 22 (3): 229-43. | Population not relevant |
| Vance JC, Boyle FM, Najman JM, Thearle MJ. Couple distress after sudden infant or prenatal death: a 30-month follow up...reprinted from J Paediatr Child Health (2002) 38:368-72. Neonatal Intensive Care, 2003; 16 (7): 45-49. | Population not relevant |
| Verdon C, deMontigny F. Experiences of Nurses Who Support Parents During Perinatal Death. J Obstet Gynecol Neonatal Nurs, 2021; 50 (5): 561-67. | Outcomes not relevant |
| Wagner T, Higgins PG, Wallerstedt C. Perinatal death: How fathers grieve. Journal of Prenatal & Perinatal Psychology & Health, 1998; 13  (2): 85-98. | Study design not relevant |
| Warland J. Caring for families when a baby dies. Australian Midwifery News, 2014; 14 (1): 24-24. | Study design not relevant |
| Watson J, Simmonds A, La Fontaine M, Fockler ME. Pregnancy and infant loss: a survey of families' experiences in Ontario Canada. BMC Pregnancy Childbirth, 2019; 19 (1): 129. | Study design not relevant |
| Weiss L, Frischer L, Richman J. Parental adjustment to intrapartum and delivery room loss. The role of a hospital-based support program. Clin Perinatol, 1989; 16 (4): 1009-19. | Study design not relevant |
| Weiss M. Care and support of women who have experienced a stillbirth. Midwives Chronicle, 1987; 100 (1195): 233-34. | Setting not relevant |
| Welborn JM. The experience of expressing and donating breast milk following a perinatal loss. J Hum Lact, 2012; 28 (4): 506-10. | Population not relevant |

| Reference | Reason for exclusion |
| --- | --- |
| Wheeler L, Fragkiadaki E, Clarke V, DiCaccavo A. ‘Sunshine’, ‘angels’ and ‘rainbows’: language developed by mothers bereaved by perinatal loss. British Journal of Midwifery, 2022; 30 (7): 368-74. | Outcomes not relevant |
| White DL, Walker AJ, Richards LN. Intergenerational family support following infant death. Int J Aging Hum Dev, 2008; 67 (3): 187-208. | Population not relevant |
| Willer EK, Droser VA, Hoyt KD, Hunniecutt J, Krebs E, Johnson JA, et al. A visual narrative analysis of children’s baby loss remembrance drawings. Journal of Family Communication, 2018; 18 (2): 153-69. | Outcomes not relevant |
| Willick ML. 'The grief never goes away': A study of meaning reconstruction and long-term grief in parents' narratives of perinatal loss. Vol. 67, ProQuest Information & Learning, 2007, pp. 6083-83. | Outcomes not relevant |
| Wilson RE. Parents' support of their other children after a miscarriage or perinatal death. Early Human Development, 2001; 61 (2): 55-65. | Setting not relevant |
| Winter GF. Breast milk donation and bereavement. British Journal of Midwifery, 2019; 27 (11): 736-36. | Study design not relevant |
| Worth NJ. Becoming a father to a stillborn child. Clin Nurs Res, 1997; 6  (1): 71-89. | Setting not relevant |
| Wright PM. Pushing on: A grounded theory study of maternal perinatal bereavement. Loyola University Chicago, 2010, pp. 157 p-57 p. | Outcomes not relevant |
| Yamazaki A. Living With Stillborn Babies as Family Members: Japanese Women Who Experienced Intrauterine Fetal Death After 28 Weeks Gestation. Health Care for Women International, 2010; 31 (10): 921-37. | Outcomes not relevant |
| Zsak ME. Personal grief and professional trauma in perinatal care: A comparative study between Italy and Hungary. Journal of Maternal- Fetal and Neonatal Medicine, 2021; 3486. | Study design not relevant |
| Zwerling B, Rousseau J, Ward K, Lo A, Harken T. Nurses experience of perinatal loss: A qualitative study on caring for patients undergoing labor induction for fetal demise or fetal anomalies. Contraception, 2020; 102 (4): 289. | Study design not relevant |
| Zwerling B, Rousseau J, Ward KM, Olshansky E, Lo A, Thiel de Bocanegra H, et al. "It's a horrible assignment": A qualitative study of labor and delivery nurses' experience caring for patients undergoing labor induction for fetal anomalies or fetal demise. Contraception, 2021; 104 (3): 301-04. | Outcomes not relevant |

Qualitative studies excluded due to methodological limitations

| Reference | Reason for exclusion |
| --- | --- |
| Agwu Kalu F, Coughlan B, Larkin P. A mixed methods sequential explanatory study of the psychosocial factors that impact on midwives’ confidence to provide bereavement support to parents who have experienced a perinatal loss.  Midwifery, 2018; 6469-76. | Methodologically weak in multiple domains |
| André B. When introduction to life becomes an introduction to death: midwifes' experiences with parents' loss of their baby in connection with birth. Nordic Journal of Nursing Research & Clinical Studies / Vård i Norden, 2000; 20 (2): 39-43. | Methodologically weak in multiple domains |
| Begley C. 'I cried ... I had to ... ': student midwives' experiences of stillbirth, miscarriage and neonatal death. Evidence Based Midwifery, 2003; 1 (1): 20-26. | Methodologically weak in multiple domains |
| Bond D, Raynes-Greenow C, Gordon A. Bereaved parents’ experience of care and follow-up after stillbirth in Sydney hospitals. Australian and New Zealand Journal of Obstetrics and Gynaecology, 2018; 58 (2): 185-91. | Methodologically weak, results weak |
| Brierley-Jones L, Crawley R, Jones E, Gordon I, Knight J, Hinshaw K. Supporting parents through stillbirth: A qualitative study exploring the views of health professionals and health care staff in three hospitals in England. European Journal of Obstetrics & Gynecology & Reproductive Biology, 2018;45-51. | Methodologically weak, a mix of methods |
| Brierly LE. Couples' experiences grieving late-term pregnancy loss in the digital era. Vol. 79, ProQuest Information & Learning, 2018. | Selection of participants unclear, results not reliable |
| Cassidy PR. The Disenfranchisement of Perinatal Grief: How Silence, Silencing and Self-Censorship Complicate Bereavement (a Mixed Methods Study). Omega - Journal of Death & Dying, 2021;302228211050500. | Analysis methods unclear, lacks information about participants |
| Dyson L, While A. Research study. The 'long shadow' of perinatal bereavement. British Journal of Community Nursing, 1998; 3 (9): 432-39. | Selection of participants unclear, results not reliable |
| Fenwick J, Jennings B, Downie J, Butt J, Okanaga M. Providing perinatal loss care: satisfying and dissatisfying aspects for midwives. Women & Birth, 2007; 20 (4): 153-60. | Methodologically weak, unclear which question is being answered and by whom. |

| Reference | Reason for exclusion |
| --- | --- |
| Gillis C, Wheatley V, Jones A, Roland B, Gill M, Marlett N, et al. Stillbirth, still life: A qualitative patient-led study on parents' unsilenced stories of stillbirth. Bereavement Care, 2020; 39 (3): 124-32. | Methodologically complicated, result is biased |
| Hutti MH, Polivka B, White S, Hill J, Clark P, Cooke C, et al. Experiences of Nurses Who Care for Women After Fetal Loss. JOGNN: Journal of Obstetric, Gynecologic & Neonatal Nursing, 2016; 45 (1): 17-27. | Methodologically weak, unclear focus, unclear what questions participants were answering |
| Kavanaugh K. Parents' experience surrounding the death of a newborn whose birth is at the margin of viability. JOGNN: Journal of Obstetric, Gynecologic & Neonatal Nursing, 1997; 26 (1): 43-51. | Methodologically weak |
| Kelley MC, Trinidad SB. Silent loss and the clinical encounter: Parents' and physicians' experiences of stillbirth-a qualitative analysis. BMC Pregnancy & Childbirth, 2012; 12  (1): 137-37. | Methodologically weak |
| King MQ, Oka M, Robinson WD. Pain without reward: A phenomenological exploration of stillbirth for couples and their hospital encounter. Death Studies, 2021; 45 (2): 152-62. | Methodologically weak |
| Lee C. ‘She was a person, she was here’: The experience of late pregnancy loss in Australia. Journal of Reproductive & Infant Psychology, 2012; 30 (1): 62-76. | Methodologically weak |
| Listermar KH, Sormunen T, Rådestad I. Perinatal palliative care after a stillbirth—Midwives' experiences of using Cubitus baby. Women & Birth, 2020; 33 (2): 161-64. | Methodologically weak, unclear if results are reliable |
| Martínez-Serrano P, Pedraz-Marcos A, Solís-Muñoz M, Palmar-Santos AM. The experience of mothers and fathers in cases of stillbirth in Spain. A qualitative study. Midwifery, 2019; 7737-44. | Methodologically weak, unclear if results are reliable |
| McCreight BS. Perinatal loss: a qualitative study in Northern Ireland. Omega: Journal of Death & Dying, 2008; 57 (1): 1-  19. | Methodologically weak, scope of the results is unclear |
| McNamara K, Meaney S, O'Connell O, McCarthy M, Greene R, O'Donoghue K, et al. Healthcare professionals' response to intrapartum death: a cross-sectional study. Archives of Gynecology & Obstetrics, 2017; 295 (4): 845-52. | Methodologically weak |

| Reference | Reason for exclusion |
| --- | --- |
| McNamara K, Meaney S, O'Donoghue K, O'Donoghue K. Intrapartum fetal death and doctors: a qualitative exploration. Acta Obstetricia et Gynecologica Scandinavica, 2018; 97 (7):  890-98. | Methodologically weak, significant risk for bias in the results, ethical concerns |
| Nurse-Clarke N. Managing Ambiguity When Caring for Women Who Experience Stillbirth. JOGNN: Journal of Obstetric, Gynecologic & Neonatal Nursing, 2021; 50 (2):  143-53. | Methodologically weak, results do not directly address the study question |
| Puia DM, Lewis L, Beck CT. Experiences of Obstetric Nurses Who Are Present for a Perinatal Loss. JOGNN: Journal of Obstetric, Gynecologic & Neonatal Nursing, 2013; 42 (3): 321-31. | Methodologically weak, results are weak |
| Pullen S, Golden M, Cacciatore J. "I'll Never Forget Those Cold Words as Long as I Live": Parent Perceptions of Death Notification for Stillbirth. Journal of Social Work in End-of- Life & Palliative Care, 2012; 8 (4): 339-55. | Methodologically weak, ethical concerns, results are weak and have significant risk for bias |
| Radestad I, Christoffersen L. Helping a woman meet her stillborn baby while it is soft and warm. British Journal of Midwifery, 2008; 16 (9): 588-91. | Methodologically weak, more of a discussion paper |
| Samuelsson M, Rådestad I, Segesten K. A waste of life: fathers' experience of losing a child before birth. Birth: Issues in Perinatal Care, 2001; 28 (2): 124-30. | Methodologically weak, results not entirely reliable |
| Sanchez NA. Mothers' perceptions of benefits of perinatal loss support offered at a major university hospital. Journal of Perinatal Education, 2001; 10 (2): 23-30. | Methodologically weak in multiple domains |
| Smith LK, Dickens J, Bender Atik R, Bevan C, Fisher J, Hinton L, et al. Parents' experiences of care following the loss of a baby at the margins between miscarriage, stillbirth and neonatal death: a UK qualitative study. BJOG: An International Journal of Obstetrics & Gynaecology, 2020; 127  (7): 868-74. | Methodologically weak, mixed population where it is not possible to distinguish which results are relevant to the target population |
| Willis P. Nurses' Perspective on Caring for Women Experiencing Perinatal Loss. MCN: The American Journal of Maternal Child Nursing, 2019; 44 (1): 46-51. | Methodologically weak, results weak |
